# Plant TDP1 (Tyrosyl-DNA Phosphodiesterase 1): A Phylogenetic Perspective and Gene Expression Data Mining

**DOI:** 10.3390/genes11121465

**Published:** 2020-12-07

**Authors:** Giacomo Mutti, Alessandro Raveane, Andrea Pagano, Francesco Bertolini, Ornella Semino, Alma Balestrazzi, Anca Macovei

**Affiliations:** 1 Department of Biology and Biotechnology ‘L. Spallanzani’, University of Pavia, via Ferrata 9, 27100 Pavia, Italy; giacomo.mutti01@universitadipavia.it (G.M.); andrea.pagano01@universitadipavia.it (A.P.); ornella.semino@unipv.it (O.S.); alma.balestrazzi@unipv.it (A.B.); 2 Laboratory of Hematology-Oncology, European Institute of Oncology IRCCS, via Ripamonti 435, 20141 Milan, Italy; francesco.bertolini@ieo.it

**Keywords:** tyrosyl-DNA phosphodiesterase, DNA damage, expression profiling, phylogenetic analysis, sequence organization

## Abstract

The TDP1 (tyrosyl-DNA phosphodiesterase 1) enzyme removes the non-specific covalent intermediates between topoisomerase I and DNA, thus playing a crucial role in preventing DNA damage. While mammals possess only one *TDP1* gene, in plants two genes (*TDP1α* and *TDP1β*) are present constituting a small gene subfamily. These display a different domain structure and appear to perform non-overlapping functions in the maintenance of genome integrity. Namely, the HIRAN domain identified in TDP1β is involved in the interaction with DNA during the recognition of stalled replication forks. The availability of transcriptomic databases in a growing variety of experimental systems provides new opportunities to fill the current gaps of knowledge concerning the evolutionary origin and the specialized roles of *TDP1* genes in plants. Whereas a phylogenetic approach has been used to track the evolution of plant TDP1 protein, transcriptomic data from a selection of representative lycophyte, eudicots, and monocots have been implemented to explore the transcriptomic dynamics in different tissues and a variety of biotic and abiotic stress conditions. While the phylogenetic analysis indicates that TDP1α is of non-plant origin and TDP1β is plant-specific originating in ancient vascular plants, the gene expression data mining comparative analysis pinpoints for tissue- and stress-specific responses.

## 1. Introduction

The story of TDP1 starts with its identification in yeast [1,2] and mammal systems [3] in the late 1990s, whereas it started to be characterized in plants only by 2010 [4,5]. It presents itself as an enzyme that can hydrolyze the covalent intermediates between topoisomerase I (Top1) and DNA [1], being included within the phospholipase D (PLD) protein family that comprises many enzymes involved in phospholipid metabolism [2]. As all PLD proteins, TDP1 is characterized by the presence of two HKD (histidine, lysine, aspartate) motives, acting as a dimer and being responsible for the catalytic activity [6,7]. When the crystal structure of the human TDP1 protein (hTDP1) was solved in 2002, it was confirmed that the two-step catalytic mechanism (specific for the PLD family) is followed with a unique approach since it possesses a chemically asymmetric active site where H263 acts as a nucleophile in the first step reaction [8]. To briefly summarize this catalytic mechanism, the first step consists of a nucleophilic attack of the Top1-DNA phosphotyrosyl bond by H263 residue (HKD motif 1), resulting in a transient covalent phosphoamide bond. Subsequently, the H493 residue (HKD motif 2) hydrolyzes the covalent intermediate using an activated water molecule and generating a product with a 3′ phosphate end that will be further processed by the endogenous DNA repair machinery [9]. Though the course of time, TDP1 had been extensively studied in human cells because of its implication in neurodegenerative disorders [10] and cancer progression [11,12]. The latter is based on the idea that TDP1 is a target for several DDR (DNA damage response) kinases [13], involved in the activation of cell cycle checkpoints, apoptosis, and DNA repair pathways [14].

In plants, the story of TDP1 begun 10 years ago when the *Arabidopsis thaliana* homologue was first identified by Lee et al. [4], who demonstrated that the AtTDP1 enzyme was able to hydrolyze the 3′ phosphotyrosyl DNA substrates related to Top1-DNA damage. The Arabidopsis *tdp1* mutants presented a different developmental phenotype characterized by dwarfism caused by a decreased number of cells, while the use of vanadate inhibited the AtTDP1 enzymatic activity and further retarded the growth of the mutant [15]. More recently, it has been demonstrated that AtTDP1 participates in the repair of DNA-protein crosslinks (DPC), as a backup pathway independent of MUS81 endonuclease and WSS1 protease [16,17]. Interestingly, in 2010, a small subfamily of *TDP1* genes was identified in the model legume *Medicago truncatula* [5]. The two genes, *TDP1α* and *TDP1β*, encode two distinct proteins both containing the highly conserved HKD domains; while the TDP1α protein has been referred to as the canonical one since it resembles most the hTDP1 organization, the TDP1β protein was shown to contain an additional HIRAN (HIP116, Rad5p, N-terminal) domain, localized between the two HKD motifs. At the time, the presence of this small subfamily of genes has been evidenced by sequence comparison also in other plant species (e.g., *A. thaliana*, *Populus trichocarpa*, *Oryza sativa*, *Triticum aestivum*) [5]. Further insights into the function of the *MtTDP1α* gene were provided by Donà et al. [18] who demonstrated that the maintenance of genome integrity in RNAi knockdown plants was severely impaired. Events like the up-regulation of senescence-associated genes, telomere shortening, impaired ribosome biogenesis, reduction in methylated cytosines, and differential transposons expression [18,19], indicate that *TDP1α* plays an essential role in plant genome stability. Additionally, even if the *MtTPD1β* gene was upregulated in the *MtTDP1α*-depleted plants, this was not sufficient to rescue the altered phenotype, suggesting that the two genes do not have an overlapping function [18]. This is supported also by the different expression patterns of the two genes observed in the model plants *A. thaliana* and *M. truncatula* in response to several types of stresses, indicating that the *TDP1β* had an immediate response while *TDP1α* is activated at a later time [20]. It has been hypothesized that this might be related to the presence of the HIRAN domain, previously predicted to function as a DNA-binding domain that recognizes several features associated with DNA damage and stalled replication forks [21]. In eukaryotes, the HIRAN domain can be found in association with other motives such as FHA (ForkHead-associated), SMARCA (SWI/SNF-related, matrix-associated, actin-dependent related chromatin), and VRR-NUC (PD-(D/E)XK nuclease superfamily) [20]. When the crystal structure of the HIRAN domain present in the human HLTF (helicase-like transcription factor) protein was solved [22], it was evidenced that the domain interacted with both single- and double-stranded DNA. The study also showed that the HIRAN domain specifically recognizes the 3′-end of DNA, indicating that it functions as a sensor of stalled replication forks and may facilitate fork regression [22]. 

Although in the past 10 years significant steps have been made to investigate the TDP1 family in plants, many questions still remain open. For instance, at which point during plant evolution this family of genes appeared? Are the differences between *TDP1α* and *TDP1β* gene expression patterns, evidenced so far in *M. truncatula* and *A. thaliana* [20], observable also in other plant species? Based on the advancements in genome sequencing, “omics” approaches, bioinformatics tools, and plant database building software, the current study aims at answering precisely these questions. A phylogenetic approach was hereby designed to investigate the origin, relation, and evolution of the TDP1 subfamily in plants. In parallel, using a collection of transcriptomics data available at ePlant [23], we have compared the expression of the two genes in different tissues, developmental stages, and stress conditions, taking into consideration archaic plants and agriculturally relevant crops. 

## 2. Materials and Methods

### 2.1. Dataset 

A total of 124 sequences were retrieved and divided into two datasets (Tables S1–S2). TDP1α and TDP1β sequences were searched on separated BLASTp (https://blast.ncbi.nlm.nih.gov/Blast.cgi) queries using as reference the sequences of *M. truncatula* [5] setting the max target sequences parameter to 5000. Then, the species of interest were manually selected. For TDP1α, 86 sequences were retrieved with the BLASTp query while the two sequences of *Pinus sylvestris* and *Taxus baccata*, not present in NCBI, were obtained by using a BLAST query in the database GymnoPlaza1.0 under the repository PLAZA4.0 [24]. The final number of sequences for TDP1α was 88 (Table S1) whereas for TDP1β 36 sequences were collected with the BLASTp method (Table S2). Fewer sequences were retrieved for TDP1β as in this case the BLASTp annotation was often not sufficiently secure.

### 2.2. Sequence Alignment 

Multiple sequence alignment (MSA) was performed on both TDP1α and TDP1β using an R function that exploits the *msa* package [25] with the ClustalOmega alignment method. This algorithm was preferred for its robustness and accuracy even when many sequences are included [26]. The alignments were converted into FASTA with the package *seqinr* [27] and the visualization was obtained using the *msaPrettyPrint* function under the *msa* library.

### 2.3. Hierarchical Clustering

Explorative relationship analyses were carried out using both Neighbor Joining (NJ) and Unweighted Pair Group Method with Arithmetic Mean (UPGMA) clustering methods using *ape* R package [28]. The distance matrixes were generated with the package *seqinr*. Modifications after visual inspection were performed using *ggtree* package [29].

### 2.4. Phylogenetic Analysis

Several phylogenetic analyses were carried out to exploit the wide range of bioinformatics tools available and as many as possible methods to unveil the evolution and the relationship between amino acid sequences.

The phylogenetic analyses on TDP1α and TDP1β proteins were carried out with MEGA X software [30] using maximum likelihood (ML) clustering method after the evaluation of the best substitution model with “Find best DNA/Protein Models” function available in MEGAX software. For TDP1α, the substitution model (lowest BIC) was set as JTT + G, where JTT refers to the Jones-Taylor-Thorton model [31] and G specifies that the rates are modelled as a gamma distribution. For TDP1β, the best model was JTT + G + I + F; the value I indicates that some sites are invariant while F indicates that the algorithm will use the observed aminoacidic frequencies. For each protein, 500 bootstrap replicates were run. Post-analyses modifications were performed with *ggtree* package as mentioned in Section 2.3.

The phylogenetic analysis of the two proteins combined was performed with mrBayes v3.2 [32] using the fixed Jones *invgamma* amino acid model, calculated for 1 million generations, sampling every 500 generations and a *burnin* of 25% for the calculation of the consensus tree. The best model was computed as before with MEGAX. In this case, because of the increased number of differences, the alignment was refined with the Gblocks server (http://molevol.cmima.csic.es/castresana/Gblocks_server.html, [33]) using the least stringent parameters. Final visualization was performed with *ggtree* package.

### 2.5. Conserved Protein Motifs

To identify the conserved motifs of TDP1α and TDP1β proteins, the multiple expectation-maximization for Motif Elicitation MEME 5.0.5 online suite software [34] was employed. After visual inspection of preliminary results, *anr* and *zoop* were the chosen options as site distribution, 6 and 7 motifs were used for TDP1α and TDP1β, respectively, while for all the other parameters the default values were maintained.

### 2.6. Gene Structure Analysis

An in-house bash script, that exploits the *Entrez-Direct* utilities [35], was used to retrieve the coding DNA sequence (CDS) from NCBI database for each protein sequence present in this study. 

The specific genetic structure (exons, introns, and untranslated regions) was evaluated with Gene Structure Display Server (GSDS) (http://gsds.gao-lab.org/, [36]) only on a subset of selected representative species.

### 2.7. Code Availability

All the codes used for the alignment, phylogenetic analyses, and conserved motives are available in the *GitHub* repository (https://github.com/giacomomutti/TDP-phylogenetic-analysis).

### 2.8. Gene Expression Data Mining

Data mining for gene expression studies was performed on the BAR Toronto eFP browser (http://bar.utoronto.ca/eplant, [23]), a visual analytic tool for exploring multiple levels of expression data in plants. Each RNA-seq data deposited in this database contains specific references to the study in which the data were generated and the type of materials, treatments, and data analyses that were used. Specifically, for gene expression data mining, we have retrieved gene expression data for the following species: *Selaginella moellendorffii* [37,38,39], *Zea mays* [40,41], *O. sativa* [42,43], *M. truncatula* [44,45], *Glycine max* [46,47], *Solanum lycopersicum* [48], and *Solanum tuberosum* [49]. The accessions of the *TDP1α* and *TDP1β* genes for the species taken into consideration are provided in Table S3. For *S. moellendorffii*, the data is provided as transcripts per million (TPM) whereas for maize the data were normalized as RPKM (reads per kilobase of transcript per million mapped reads) and for all other indicated species the data is FPKM (fragments per kilobase of exon model per million reads mapped)-normalized. The selected mode for all the species was “absolute,” indicating the expression levels for each type of sample/condition is directly associated with the most intense signal recorded for each gene. The intensities obtained through eFP (electronic fluorescent pictography) are normalized using two different methods: RMA and MAS5. RMA (robust multichip analysis) is an algorithm that uses a set of chips (>3) which are assumed to have the same background noise and ignore mismatches, while MAS5, selected for the data in the current investigation, can be used with a single chip and isolates individual signals by removing the background noise [50]. In all the studies, samples were analyzed in triplicates while only the mean values were retrieved and used to represent the data as heatmap models generated using the Shinyheatmap (http://shinyheatmap.com/) application [51]. Within this application, the expression values are represented as Z-score, a numerical measure that describes the relationship of a value to the mean of a group of values.

## 3. Results 

### 3.1. Phylogenetic Analyses Show that TDP1β Appeared in Ancestral Plants

To construct the phylogenetic tree of the plant TDP1 family, several approaches based on multiple sequence alignments were tested and are presented in this work. The final alignment of TDP1α consists of a total of 1384 amino acids of which only 12 are perfectly conserved in all the 88 species taken into consideration (Figure S1). Notably, among the 12 residues, three (HH-K) belong to HKD motif 1; the amino acid found between the catalytic histidine and lysine was serine in plants and threonine in animals, *Saccharomyces cerevisiae*, and *Physcomitrella patens* (bryophyte). The H and K residuals of the HKD motif 2 are highly conserved, except for pineapple (*Ananas comosus*) and clementine (*Citrus clementina*) (Figure S1). The final alignment of TDP1β was more conserved when compared to the one of TDP1α. In a total of 1523 amino acids, 131 residues are present in all the 27 species considered, including the H and K residues of HKD motif I and motif II (Figure S2). The number of sites highly conserved (present in more than 90% of the organisms considered) is higher in TDP1β (17.6%) than TDP1α (10.9%). This may be likely attributable to the presence of very distant sequences such as animals and fungi in the TDP1α alignment (Figure S3). 

Hierarchical clustering, namely Unweighted Pair Group Method with Arithmetic mean (UPGMA) and Neighbor Joining (NJ), of the two alignments was initially implemented to perform a preliminary inspection of the relationship among the considered species (Figures S4 and S5). These analyses showed that the TDP1α sequences from fungi, animals, algae, ancestral plants/gymnosperms are grouped together. For TDP1β, *S. moellendorffii* acts as an outgroup. This plant is a lycophyte, one of the most ancient groups of vascular plants, and shows the presence of both TDP1α and TDP1β sequences. In the successive node, all the monocots diverge from the eudicots and *Amborella trichopoda*, a basal angiosperm.

**Figure 1:**
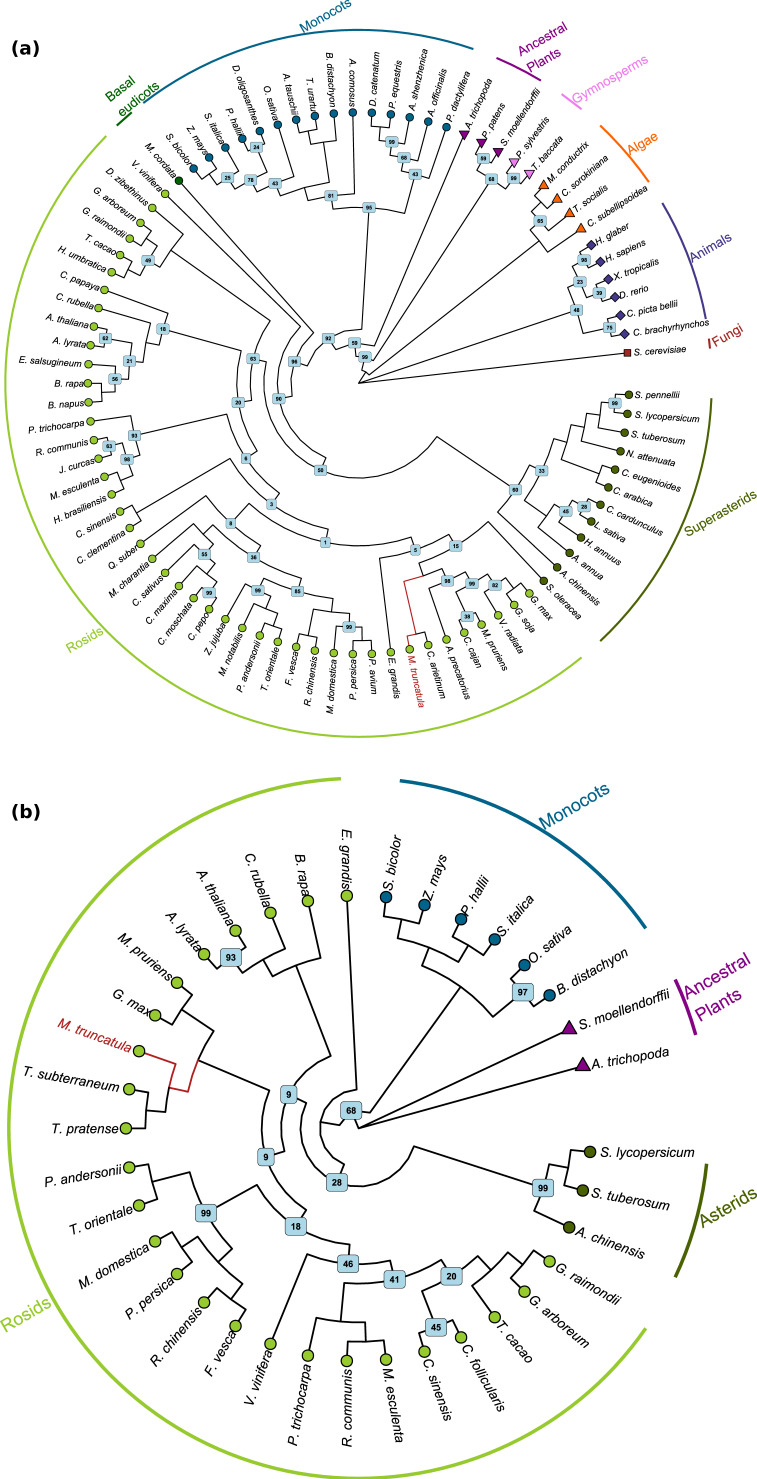
Phylogenetic tree constructed based on the (**a**) TDP1α and (**b**) TDP1β sequences. For TDP1α, 500 bootstrap maximum likelihood tree of 88 protein sequences was used while 36 protein sequences are representative for TDP1β. Bootstrap values are shown only if lower than 100. The different clades are marked with different point and bar colors. *M. truncatula* branch is highlighted in red.

To better understand the evolutionary relationship between the species present in our dataset based on the two TDP1 sequences, a maximum likelihood (ML) analysis was performed. From the 88 sequences of the TDP1α, the tree built (Figure 1a) reveals once again, the outgroup position of *S. cerevisiae* together with the animals. Algae is the first clade to be separated from the outgroups followed by the two gymnosperms, (*T. baccata* and *P. sylvestris*) and ancestral vascular plants (*S. moellendorffi* and *P. patens*), which confirm *A. trichopoda* as the most basal lineage of the clade of the angiosperms [52]. In the monocots group, a division from commelinids and lilioid is observed, except for date palm (*Phoenix dactylifera*), a species belonging to the commelinids clade, whose TDP1α seems to resemble the one of the lilioids. *Macleaya cordata*, a basal eudicot species belonging to the *Ranunculales* order, is placed as an outgroup of all the core eudicots species, a result supported by literature reporting the analysis of its genome [53]. The results of the ML analysis shows that the core eudicots are well separated in the two superclades of rosids and superasterids with only the exception of two species, common grape (*Vitis vinifera*) and spinach (*Spinacia oleracea*) belonging to the order of the *Vitales* (the most basal clade in the rosids) and *Caryophillales* (a clade of the superasterids), respectively. If the first one is well separated from all the other clades, the *S. oleracea* TDP1α is more similar to the rosids than to superasterids. However, few basal nodes in the *Rosids* clade have very low support values, whereas more recent nodes seem to be better explained. *M. truncatula* is placed in the clade of *Rosids* in a cluster including all the species present in the dataset belonging to the *Fabaceae* family. The grouping within this family is well supported. This family has been confirmed to be monophyletic by different molecular analysis [54]. However, the sister groups of this node contain *Eucalyptus grandis* and *S. oleracea*, two very distant species with respect to *Fabaceae*. 

The evolutionary relationships observed in TDP1β ML tree have a high level of similarity with the one of TDP1α (Figure 1b). The new outgroup, only consisting of the two ancestral plants, is different from TDP1α because of the lack of this protein in fungi, algae, and animals. Also, in this case, monocots and eudicots clades are the first to diverge after the outgroup, and, among the eudicots clade, it is possible to notice the division between rosids and asterids. *M. truncatula* is once again clustered in the family of *Fabaceae* together with other available species. These trees are both represented with the corresponding multiple sequence alignment in Figures S6 and S7.

**Figure 2:**
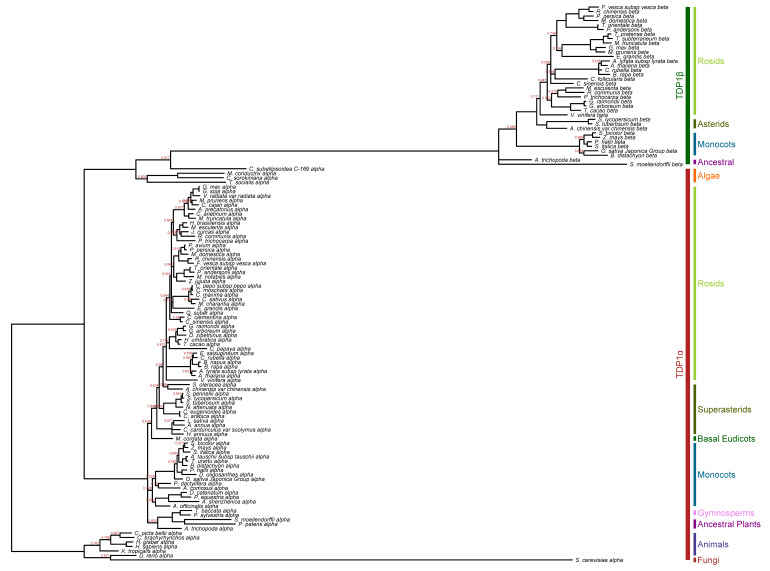
Bayesian phylogenetic tree of combined TDP1α and TDP1β protein sequences. The tree is rooted for animals and *S. cerevisiae* TDP1α sequences. Posterior probabilities (p.p.) are shown only if <1. The different sequences and clades are annotated with different bar colors.

A Bayesian inference phylogenetic analysis (Figure 2, Figure S8) of all the sequences present in the used dataset reveals two major clades belonging to the two TDP1 proteins, except for the TDP1α algae species which groups with the TDP1β species (posterior probability—p.p. = 0.92). Within the major clades, the relationships observed in ML trees are generally supported, with some differences in nodes that were supported by a low number of bootstraps. 

The node comprising all TDP1α plant species is not resolved, trifurcating in ancestral plants/gymnosperms, monocots, and eudicots. *Asterids* species are divided into *Asterales* at the base and *Solanales/Gentianales* which are merged with the *Rosids* (p.p. = 0.65). Spinach (*S. oleracea*) and kiwi (*Actinidia chinensis*), even though they represent basal species of Superasterids, are also grouped with the *Rosids* (p.p. = 0.58). The *Rosids* node is not resolved, as indicated by the low bootstrap values previously found (Figure 1b, Figure S7), and grape (*V. vinifera*) acts as an outgroup. *Brassicales* are grouped together, except for papaya (*Carica papaya),* which groups with *Malvales* species (p.p. = 0.77), differently from the ML tree. As in the ML tree, all *Fabales* are clustered together with high confidence and again *M. truncatula* seems to be near the chickpea (*C. arietinum*). All TDP1β sequences share the same node with TDP1α algae sequences, with *Coccomyxa subellipsoidea* being the closest. However, their respective branches are much longer than the others. Both the ancestral plants act as outgroup even if this node is not resolved. Monocots and eudicots are correctly divided (p.p. = 0.97) like *Asterids* and *Rosids* species (p.p. = 1). *V. vinifera* sequence is at the basis of the *Rosids* clade, differently from the ML tree. *Malvales* and *Malpighiales* are grouped together with high confidence (p.p. = 0.98). Then, another polytomy is present, including among the other *Brassicales*, *Rosales*, and *Fabales* sequences. *E. grandis* is the sister group of the *Fabales*, whereas in the ML tree it was basal to all eudicots species. Overall, lower nodal support (mean of node support) is recorded in TDP1α compared to TDP1β. 

The combined Bayesian phylogenetic analysis revealed that while the TDP1α is of non-plant origin, the TDP1β is confirmed to be plant-specific and postulate its origin between the algae TDP1α and the TDP1β vascular ancestral plants.

### 3.2. Similarities and Differences within the TDP1α and TDP1β Protein and Gene Structures

**Figure 3:**
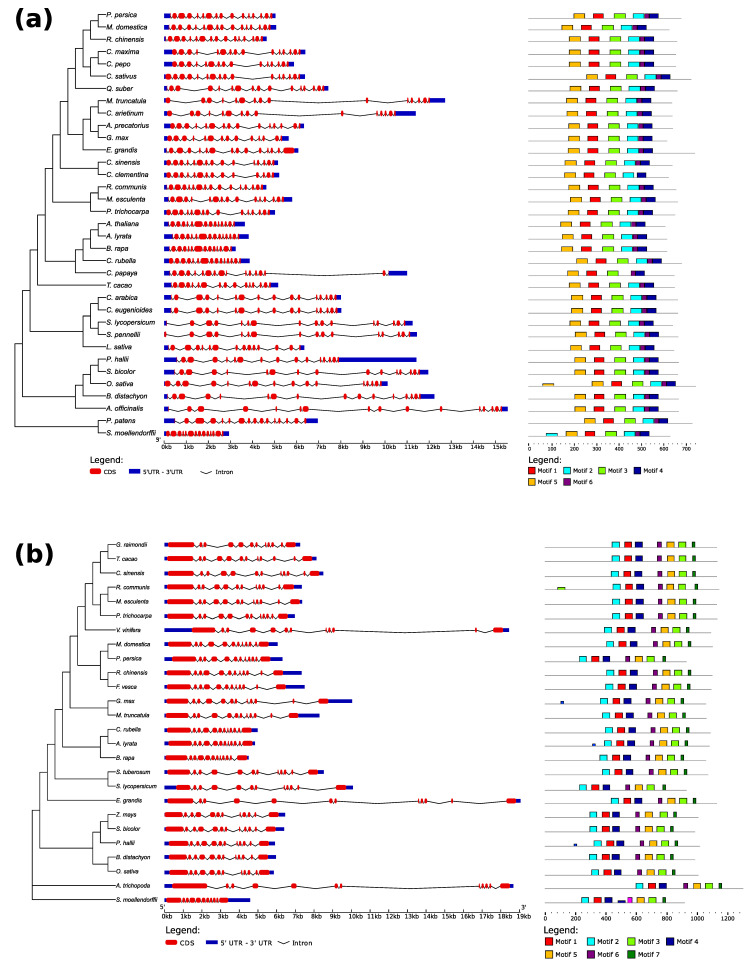
Gene and protein structure of selected (**a**) TDP1α and (**b**) TDP1β sequences. In the gene structure (central part of the figure), UTRs are represented as blue boxes, introns as black lines, and exons as blue round boxes. The colored boxes in the right part of the figure are recurring motifs found with MEME5.0 in the protein sequences (Tables S4 and S5). Lower boxes represent lower confidence. The phylogenetic tree on the left is the same as in Figure 1, trimmed for a selected subset of species.

We applied gene structure analysis to uncover differences at the genetic level within the clusters previously identified. It is possible to observe that the structure of *TDP1α* gene is very similar among the Brassicaceae cluster (*A. thaliana*, *A. lyrata*, *C. rubella*, *B. rapa*) or the Cucurbitaceae (*Cucumis sativus*, *C. maxima*, *C. pepo*), characterized by many short introns (Figure 3a). On the other hand, for the well-known family of Fabaceae, to which *M. truncatula* belongs, the gene structure is quite diverse. In particular, *M. truncatula* shares long introns with the *C. arietinum*, not present in the other two species of this group (*A. precatorius*, *G. max*). The genes of monocots species are generally longer than the eudicots because of long introns. Moreover, a high level of conservation along all the species present in this analysis was observed from the six protein motifs identified by MEME5.0 analyses (Table S4), in which the HKD is represented by motif 1 (red) and HKD-II by motifs 4 (blue) and 6 (purple). All sequences present these motifs in the same order and numerosity, except for *O. sativa* and *S. moellendorfii* which have one extra motif 5 (orange) and one extra motif 2 (cyan), respectively. However, these two motifs have lower confidence than the others (Figure 3a).

The gene structure on TDP1β (Figure 3b) revealed a higher level of conservation among all the species compared to TDP1α. High differences were only observed in the structure of four species, *S. moellendorfili*, *V. vinifera*, *A. trichopoda*, and *E. grandis* which contain long introns. Among the Fabaceae, *M. truncatula* has a structure highly similar to G. max and it is quite different from the one of the other species. The protein motif analysis evidenced the presence of seven conserved motifs for the TDP1β protein along with the recurrence of HKD-I motif as motif 4 (blue) and HKD-II as motif 7 (dark green). The HIRAN domain is represented as recurrent motif 6 (purple) (Figure 3b, Table S5). 

Overall, the TDP1α and TDP1β protein and gene structure analyses evidence similarities given the conservation of the HKD domains and sequence length distinctions for monocots and dicots, while pinpointing the specific presence of the HIRAN domain in TDP1β. 

### 3.3. Expression Profiles of TDP1α and TDP1β in the Archaic Vascular Plant S. moellendorffii

Based on the data obtained from the phylogenetic analysis, *S. moellendorffii* is the first plant that shows the presence of both *TDP1α* and *TDP1β* genes. This ancient vascular plant, member of lycophyte family, had been observed in the fossil record around 410 million years ago and is regarded as an important model organism for comparative evolution genomics [55]. A recent publication evidenced transcriptional gene modules that indicate duplication events, at least related to the biosynthesis of the cell wall and secondary metabolism [56]. 

**Figure 4:**
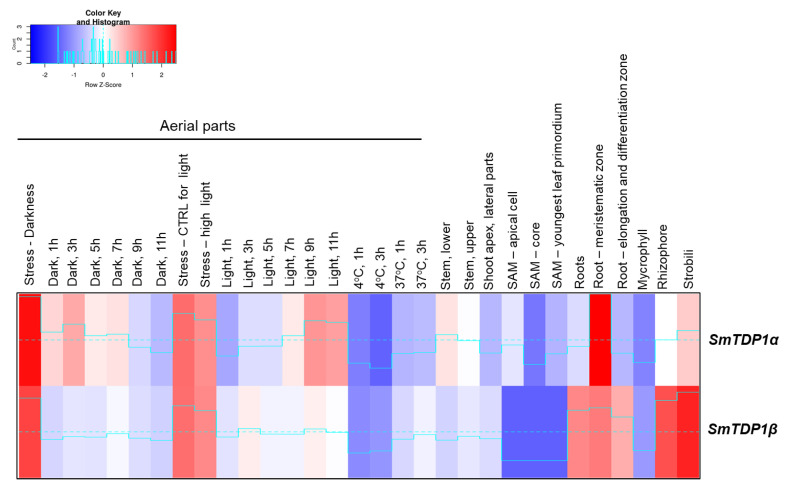
Expression profiles of the *TDP1α* (Smo170833) and *TDP1β* (Smo415650) genes in *S. moellendorffii.* Data was collected from http://bar.utoronto.ca/efp_selaginella/cgi-bin/efpWeb.cgi and heatmaps were generated using the Shinyheatmap (http://shinyheatmap.com/) application.

The gene expression data, recovered from BAR, refers to experiments carried out to study the diurnal cycle [37], root meristems [38], and shoot meristems [39]. The generated heatmap (Figure 4) evidences the expression patterns for the *SmTDP1α* (Smo170833) and *SmTDP1β* (Smo415650) genes in the considered samples/conditions. The highest expression levels of *SmTDP1α* are revealed in the root meristematic zone while for the *SmTDP1β* this is observed in strobili. Regarding the data related to the diurnal cycle, it is possible to observe that both genes are differentially expressed after several hours of treatment, with *SmTDP1α* generally showing higher expression values than *SmTDP1β* while both genes are most expressed in control experiments as well as total darkness and high light exposure. Contrasting temperatures (4 °C and 37 °C) seem to not activate the response of the genes. 

Overall, these data show a prevalence of similitudes in the two gene expression patterns, with distinct differences only in few tissues (e.g., strobili and rhizosphore). 

### 3.4. Expression Profiles of TDP1α and TDP1β in Monocots 

To look into the expression patterns of the two genes in monocotyledonous plants, we have mainly focused on rice (*O. sativa*) and maize (*Z. mays*) as these are cereal crops that have an overwhelming agricultural and economic importance. 

**Figure 5:**
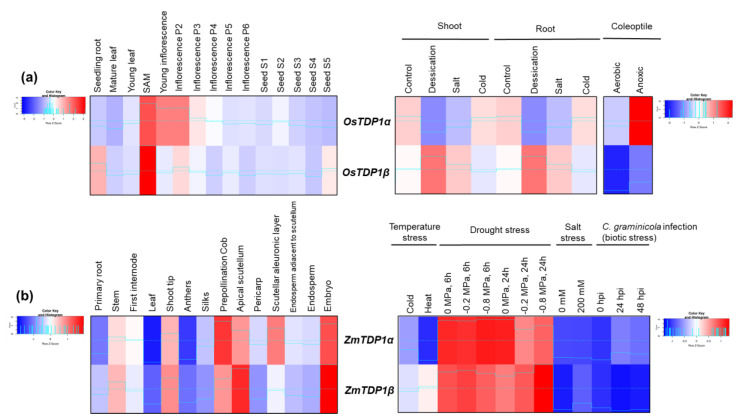
Expression profiles of the *TDP1α* and *TDP1β* genes in cereal crops. (**a**) *O. sativa.* (**b**) *Z. mays.* Expression data from different tissues and stress conditions are given in heatmaps generated based on Z-score values at http://shinyheatmap.com/.

Data on rice tissue and stress conditions were mainly retrieved from the study of Jain et al. [42]. Tissues were collected from *O. sativa* subsp. indica var. IR64 grown under greenhouse conditions while for stress treatments, 7-days-old seedlings were used as follows: salt stress—200 mm NaCl solution for 3 h, desiccation—seedlings dried for 3 h between folds of tissue paper at 28 °C ± 1 °C, cold—4 °C ± 1 °C for 3 h. Data on anoxia were retrieved from Lasanthi-Kudahettige et al. [43], who used *O. sativa* subsp. japonica var. Nipponbare, hydroponically grown 20-day-old plantlets under air/anoxia treatments (dark, 28 °C). The expression pattern of *OsTDP1α* (LOC_Os07g34598) and *OsTDP1β* (LOC_Os04g33050) genes show similar profiles in plant tissues with both genes being highly expressed in the shoot apical meristem (SAM). High expression levels of *OsTDP1α* gene are observed also in young inflorescence tissues while for *OsTDP1β* relatively high gene expression is observed in seedling roots (Figure 5a). Interesting data are available when looking at gene expression during stress treatments, where contrasting responses can be seen; while the *OsTDP1β* gene is more expressed during desiccation and salt treatments, the *OsTDP1α* gene is far less expressed under the same conditions. Differently, the anoxic treatments triggered an elevated expression in *OsTDP1α* and low levels of *OsTDP1β* (Figure 5a).

However, this type of behavior is not maintained also in maize (Figure 5b) where the responses of the two genes are similar under biotic (*Colletotrichum graminicola* infection causing the anthracnose of maize disease) and abiotic (temperature, drought, salt) stresses. While the genes are highly expressed under different osmotic pressures (−0.2 and −0.8 MPa after 6 and 24 h of exposure), their expression levels are low under the rest of the imposed treatments. These data were collected from the maize developmental gene atlas that reflects major transcriptional profiles involved in abiotic or biotic stress as well as in plant development [40,41]. This trend seems to be maintained also in developmental tissues, with few variations; *ZmTDP1α* (Zm00008a028998) highest expression is present in prepollination cob while for *ZmTDP1β* (Zm00008a007514) this is evident in the apical scutellum. At the level of the scutellar aleuronic layer, the expression of *ZmTDP1α* gene is prevalent. 

Summarizing, the expression patterns of *TDP1α* and *TDP1β* genes in monocots, namely rice and maize, indicate species-specific behaviors. 

### 3.5. Expression Profiles of TDP1α and TDP1β in Dicots 

To investigate the expression patterns of *TDP1α* and *TDP1β* genes in dicotyledonous, we focused on agriculturally relevant families of plants, namely *Solanaceae* and *Fabaceae* (or *Leguminosae*). The model plant *A. thaliana* was not included in this analysis for several reasons; first, we wanted to focus especially on crops to evidence the applicability side of this basic research, and second, because the Arabidopsis database is much more extensive, containing a big amount of information that could be used as stand-alone. 

**Figure 6:**
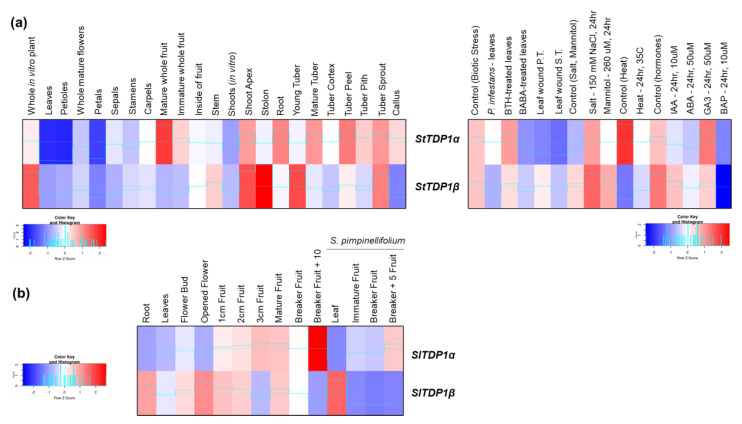
Expression profiles of the *TDP1α* and *TDP1β* genes in cereal crops. (**a**) *S. tuberosum.* (**b**) *S. lycopersicum and S. pimpinellifolium wild relative.* Expression data from different tissues and stress conditions are given in heatmaps generated based on Z-score values at http://shinyheatmap.com/.

Within the *Solanaceae* family, we investigated the gene expression patterns in *S. tuberosum*, *S. lycopersicum*, and its wild relative *S. pimpinellifolium* (Figure 6). The data on potato were retrieved from a transcriptome study that evaluated gene expression profiles in major organs, developmental stages, and stress-related conditions, using the double monoploid *S. tuberosum* Group Phureja (clone DM1-3 516R44) [49]. Developmental tissues from vegetative and reproductive organs were collected from greenhouse-grown plants whereas shoots and roots from plants grown in vitro were also considered. A *Phytophthora infestans* inoculum (Pi isolate US8: Pi02-007) was used to induce biotic stress along with other conditions such as wounded leaves to mimic herbivory attack and use of the known elicitors of the immune response, acibenzolar-s-methyl (BTH, 100 µg/mL) and DL-β-amino-n-butyric acid (BABA, 2 mg/mL). For the induction of abiotic stresses, plants were grown in vitro and treated for 24 h with heat (35 °C), salt (150 mM NaCl), and mannitol (260 µM). Additionally, different plant hormones, such as abscisic acid (ABA, 50 µM), indole-3-acetic acid (IAA, 10 µM), gibberellic acid (GA3, 50 µM), and 6 benzyl amino purine (BAP, 10 µM) were used for hormone-induced stress. When considering the expression of *StTDP1α* (PGSC0003DMG400024073) and *StTDP1β* (PGSC0003DMG400014226) genes in plant organs, a differential, tissue-specific pattern is observed (Figure 6a). *StTDP1α* is highly expressed in mature fruits and tubers while a low expression is present in leaves, petioles, and petals. Differently, *StTDP1β* is highly expressed in stolon, young tubers, shoot apex, and whole in-vitro-grown plants. Looking into the stress conditions, both genes are downregulated by biotic stresses and hormone treatments and upregulated by osmotic stresses. On the other hand, while *StTDP1α* is downregulated also during heat stress, the *StTDP1β* gene is upregulated under these conditions. 

Less data are available for tomato plants. The data hereby retrieved come from the study of Koenig et al. [48] which deals with comparative transcriptomics in domesticated (*S. lycopersicum* var. M82) and wild tomato (e.g., *S. pimpinellifolium*), mainly related to the developmental context. The tested materials consisted of roots, leaves, flowers, young green fruits, and mature fruits collected from plants grown under greenhouse conditions. The generated heatmap (Figure 6b) shows that *SlTDP1α* (Solyc03g031820.2) most intense expression is in fruits whereas for *SlTDP1β* (Solyc03g117960.2) is mostly expressed in roots and flowers. Hence, the tissue-specific gene expression variation observed for potato plants is maintained in tomatoes as well. 

**Figure 7:**
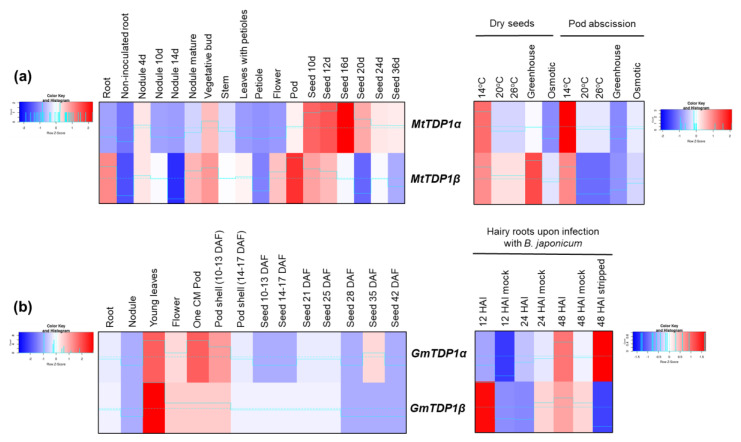
Expression profiles of the *TDP1α* and *TDP1β* genes in cereal crops. (**a**) *M. truncatula.* (**b**) *G. max.* Expression data from different tissues and stress conditions are given in heatmaps generated based on Z-score values at http://shinyheatmap.com/.

Within the *Fabaceae* family, we focused on *M. truncatula* (barrel medic), an important model species for legume research as well as a relevant forage crop for the Mediterranean and semi-arid areas, and *G. max*, widely grown for its edible beans (Figure 7). For *M. truncatula*, data regarding transcriptomics in all major organ systems were retrieved from Benedito et al. [44] while data on the effect of stress (temperature, osmotic) on dry seeds and during pod abscission were recovered from the study of Righetti et al. [45]. In the latter case, the imposed conditions were as follows: standard conditions (20/18 °C), low temperature (14/11 °C), high temperature (26/24 °C), osmotic stress (20/18 °C; −0.1 MPa), and greenhouse conditions (variable temperature and light). Within the different plant organs taken under consideration, *MtTDP1α* (Medtr7g050860) gene is highly expressed at the early stages of seed development, while *MtTDP1β* (Medtr8g095490) gene is most expressed in roots, mature nodules, vegetative buds, flowers, and pods (Figure 7a). As for the effects of stress, both genes are upregulated by cold and downregulated by osmotic conditions, especially in dry seeds. 

The situation is relatively different for soybean (Figure 7b) as, in this case, both genes are expressed at low levels during seed development and at high levels in young leaves, flowers, and pods. Data deposited in BAR came from the RNA-seq soybean transcriptomic study of Severin et al. [47] for the different tissues while data for root hair cells in response to *Bradyrhizobium japonicum* (USDA110) infection were reported by Libault et al. [46]. The seeds were inoculated with *B. japonicum* cell suspension or water (mock inoculation) after 3 days of germination (under sterile conditions). The Illumina transcriptome data were compared between inoculated and mock-inoculated root hair cells harvested at 12, 24, and 48 h after inoculation (HAI), as well as stripped roots (roots devoid of root hairs), harvested at 48 HAI. Comparing mock with inoculated samples at the different time points, it is observed that the *GmTDP1β* (Glyma.08G005800) gene is highly induced after 12 h of inoculation while the *GmTDP1α* (Glyma.04G064300) gene is upregulated after 48 h. The high expression of *GmTDP1α* gene is maintained also in the roots devoid of root hairs (stripped) at 48 h after inoculation. So, in this case, the differential expression of *GmTDP1* genes upon *B. japonicum* infection is time-dependent. 

Overall, the results gathered from dicot species indicate a tissue-specific expression, in the case of potato, tomato, and barrel medic, as well as a time-dependent behavior as in the case of soybean response to biotic infections. 

## 4. Discussion

For the first time, the current study investigated the evolutionary origin of the *TDP1* gene family in plants alongside explorative RNA-seq data mining to provide further insights into the function of the two genes. As evidenced by the literature in mammalian and plant kingdoms, the *TDP1* genes encode important proteins assigned to DNA damage repair biological processes. Hence, because of the essential role that DNA repair has in preserving genome integrity and in plant defense against both biotic and abiotic stresses, the study of the TDP1 origin and gene expression data provide additional support for potentially developing highly tolerant plants. 

To understand when the two sequences appeared from an evolutionary perspective, a phylogenetic approach was implemented. The methodologies applied in this study are in line with other recent publications investigating the phylogenetic relations in plant gene families [57,58,59,60]. Our evolutionary analyses reveal that the phylogenesis of the TDP1 protein subfamily broadly overlaps the taxonomic division, often clustering together species belonging to the same clade, order, and family. This division is observed for both proteins and a high degree of similarity is found when the topology is compared. TDP1α outgroup comprised also protein sequences of non-plant species, suggesting a non-plant origin of this specific isoform. Therefore, we suggest a putative origin of the TDP1α plant protein in the non-plant kingdom, likely in the last common ancestor between plants and opisthokonts (animal and fungus kingdom). Moreover, from our results and from the sequences available in our dataset an α origin of the β sequence is proposed, and precisely it is placed in between the TDP1α of unicellular green alga *C. subellipsoidea* and the TDP1β of the ancestral vascular plants *S. moellendorfili*, and *A. trichopoda*. The appearance of this sequence may have happened based on its likely important role in the adaptation to a harsh environment, with extremely low temperatures and low atmospheric humidity to a more temperate environment. Indeed, all the plant species that carry the TDP1β are *Tracheophytae* and recently has been shown that this node is not characterized by a great number of novel genes, oppositely to the *Embryophyta* node (including the bryophyte *P. patens*) that is regarded as one of the most drastic transition in plants evolution [61]. Nonetheless, the novel genes acquired during the vascular adaptation are enriched in the defense response to biotic (bacterial infection) and (water and salt) abiotic stresses [61]. So, our results indicate that TDP1β is specific to plants, and believed to have appeared ~410 million years ago at the divergence into euphyllophytes and lycophytes lineages [62]. *A. trichopoda* is considered as the single sister species to all angiosperms. The Amborella Genome Project [63], revealed the presence of an ancient genome duplication event previous to angiosperm diversification, but no specific evidence of lineage-specific genome duplications was seen. The Consortium also identified new gene families (mostly involved in floral development) and gene duplications, presuming that a whole-genome duplication event preceded the evolution of this ancestral angiosperm. On the other hand, the sequencing of the *S. moellendorffii* genome indicated that the transition from non-seed vascular to flowering plants required the acquisition of a considerable number of new genes, mainly related to developmental pathways (e.g., meristems, hormone signaling, and biogenesis) [55]. 

The HKD motifs are extremely conserved, especially regarding the catalytic amino acids, with a notable difference in the amino acids between the fundamental H-K sites in TDP1*α* HKD-I motif in *P. patens* which is identical to the one in animals and *S. cerevisiae*. These motifs are present in almost all the species included in our analysis, except for TDP1*α* HKD-II motif in *A. comosus* and *C. clementina.* These exceptions, here recorded for the first time, may be related either to an ancestral residual in the case of *P. patens* or to a different environmental adaptation and evolutionary selective pressures [61]. The *TDP1α* gene structure analysis shows that there is a great difference between species belonging to the *Fabaceae* clade. The main difference follows the distinction between millietioid legumes (*Glycine* and *Abrus*) and IRLC (Inverted Repeat-lacking clade) represented by *Medicago* and *Cicer*. These clades are characterized by an ancestral WGD (Whole Genome Duplication) that may have caused genomic rearrangements [64,65]. Also, papaya has a differentiated structure from all other *Brassicales* species. This species genome is three times the size of the *Arabidopsis* genome, although containing far fewer genes supposedly due to the lack of recent duplication events [66]. Differently, the *TDP1β* gene structure is more conserved (except for *E. grandis*, *V. vinifera*, and *A. trichopoda*). *E. grandis* is a species belonging to the *Myrtales* order which is characterized by a lineage-specific WGD [67]. *V. vinifera* genome is characterized by a considerably lower number of genes with respect to the close *P. trichocarpa* [68]. Finally, *Amborella*, despite being the sister lineage to all extant flowering plants, shows no evidence of lineage-specific duplications or transposons insertions [63]. A recent publication investigating the PLD family of proteins, to which TDP1 belongs, has revealed the importance of multiple conserved motifs that may have different regulatory and catalytic functions [69]. One common feature found in the plant PLDα isoform, along with other PLDs from microbes and eukaryotes, was the duplicated catalytic HKD motif, found as well in TDP1α and TDP1β. Among the different organisms investigated in this study, the authors reported some initial anomalies present in algal sequences (e.g., the presence of an unorthodox HKS motif), which were however dismissed upon further analyses, concluding that some of the deposited sequences are unreliable [69]. The origin of plant PLDα still needs to be clarified as well, since green algae do not seem to possess this sequence, with few exceptions (*C. subellipsoidea*, *Phaeodactylum tricornutum*) hypothesized to be the result of a horizontal transfer from microbes [70]. As for the TDP1β sequence, this is quite a unique combination considering the presence of a HIRAN domain between the two HKD motifs. Phylogenetic studies into the distribution of the HIRAN domain demonstrated that it was acquired early in the evolution of eukaryotes from bacteria or phages, whereas the presence of the HIRAN domain in TDP1 is unique to plants [21]. Nonetheless, an evolutional analysis of the domain architecture of all HIRAN domain-containing proteins indicated that it is most prevalent in eukaryotes and predominantly coupled with SWI2/SNF2 helicase-like and RING-finger domains [71]. Taken together, all these studies, including the current one, strengthen the need to further investigate this unique association of HKD-HIRAN-HKD organization present in the plant TDP1β sequence, along with the function of this gene/protein.

**Figure 8:**
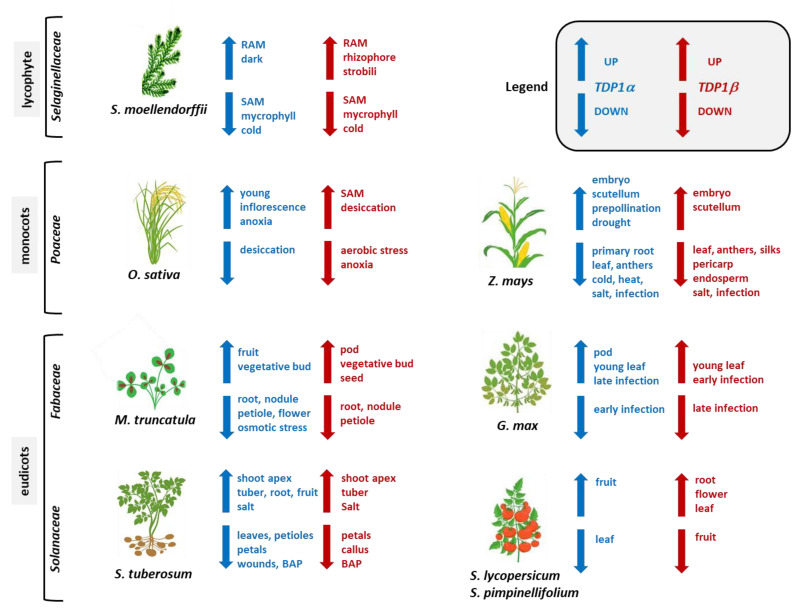
Schematic representation of *TDP1α* and *TDP1β* gene expression patterns in representative lycophytes, monocots, and dicots. *TDP1α* expression is given in blue while *TDP1β* is given in red. Arrows indicate increased or decreased accumulation, respectively.

As evidenced by the cited literature, our interest in plant TDP1 started 10 years ago when we first identified the presence of the *TDP1α* and *TDP1β* genes in the model legume *M. truncatula* [5]. Since then, we have demonstrated the crucial role that the *TDP1α* gene plays in maintaining genome integrity [18,19] as well as the differential expression of the two genes in response to different stress conditions [5,72,73], especially the early temporal responsiveness of *TDP1β* compared with *TDP1α* observed in both model species *A. thaliana* and *M. truncatula* [20]. Taking advantage of the increasing amount of data derived from RNA-seq analyses and elegantly stored in dedicated databases, such as BAR (BioAnalytic Recourse), in this work we extracted the data relative to these two genes and compared their responses in different tissues, organs, developmental stages, and stress conditions in several species. Figure 8 gives a schematic representation of the observations made based on the mined data. Relevant information that derives from the results gathered from *S. moellendorffii* is the high expression of *TDP1α* and *TDP1β* in root apical meristem (RAM) concomitant with low expression in shoot apical meristem (SAM). Conversely, in higher plants (monocots and dicots) the genes seem to be more expressed in SAM, shoot apexes, or vegetative buds. Transcriptomics studies have been conducted to investigate the differential gene expression patterns and the origin of both root [38] and shoot [39] development. An extensive comparative analysis of gene expression patterns during root formation carried out in seven plant species (*A. thaliana*, *S. lycopersicum*, *G. max*, *C. sativus*, *O. sativa*, *Z. mays*, and *S. moellendorffii*), pinpoints that despite the considerable variation in the size and cellular anatomy of roots in these species, the root developmental program is well conserved [38]. On the other side, the transcriptomic analysis of SAM collected from *Equisetum arvense* and *S. moellendorffii*, and compared to maize, indicates the presence of independent molecular genetic programs for shoot development [39]. By conducting an interspecies heatmap comparison of SAM to visualize the relative transcript accumulation of genes involved in meristem function, the authors evidenced several transcriptional regulators, hormone-related genes, components of small RNA biogenesis, and DNA repair, that are all up-regulated in angiosperm SAMs [39]. Both SAM and RAM contain specific regions where subpopulations of stem cells (the progenitors of all cells that make up the entire plant body and presumed offspring) are present, and that these stem cells niches (e.g., root quiescent center, QC) are characterized by slower rates of division compared to the rest of the cells. Importantly, it is rather critical to maintaining the genome integrity of this fraction of cells [74], and, for this reason, these cells are more resistant to DNA damage [75], probably because of their slow-paced cell division. For instance, in the event of stem cell loss as a result of DNA damage, Arabidopsis QC cells seem to be engaged in a cell division program to supply the lost stem cells, thus enabling rapid recovery of root growth following plant transfer from DNA stressed to non-stressed conditions [75]. To this purpose, high expression levels of *TDP1* genes in RAM may as well contribute to the proper maintenance of genome integrity in important cell stem niches. 

Besides the important role that DNA repair has during plant development, for instance as shown by the presence of specific DNA stress checkpoint controls [76], it is well ascertained that different types of stress conditions induce DNA damage and DNA repair pathways are activated to counteract it [77,78]. In this context, the function of TDP1 was associated with the base excision repair (BER) pathway [18,79], as well as with the repair of DNA-protein crosslinks [16,17]. In this work, concerning the response of the *TDP1* genes to abiotic stress conditions, two distinct patterns can be discussed: (1) a similar behavior of the genes in response to osmotic and temperature-related stresses (e.g., salt, cold); and (2) opposing behavioral patterns in response to desiccation and anoxia. Under biotic stress, it is possible to observe a decreased expression of both genes in maize whereas a more interesting situation is evident in soybean where a time-dependent differential expression is encountered. In this latter case, early induction of *TDP1β* and late induction of *TDP1α* is observed. As already mentioned, this type of time-dependent behavior was evidenced in *M. truncatula* and *A. thaliana* under different types of abiotic stresses [20]. Looking into the contrasting response of the two genes under different stress conditions, very interesting is the case of rice exposed to desiccation stress or anoxia. While *TDP1α* is highly expressed under anoxic conditions, *TDP1β* is highly expressed under desiccation conditions. Transcriptomics studies of rice grown under anoxia revealed that genes coding enzymes that need oxygen for their activity were substantially downregulated [43]; the authors illustrate this as an adaptive mechanism to prevent the production of enzymes that cannot operate under anoxia and save the energy for the activation of the required responsive pathways. Hence, it may be hypothesized that the *TDP1β*, unlike *TDP1α*, may require the presence of oxygen for its function. Another interesting article discusses the cross-tolerance effects of anoxia and desiccation caused by heat in Arabidopsis [80]. The results indicated that pretreatments with hypoxia or heat resulted in better anoxia tolerance but anoxic pretreatment did not result in tolerance to heat stress; in this particular case, the reasoning behind this behavior involves a differential activity of heat shock protein (HSP)-coding genes [80,81]. In *M. truncatula* desiccated (dry) seeds, we observed a considerably higher expression of *TDP1β* compared to *TDP1α* while during seed rehydration *TDP1α* is more expressed than *TDP1β* [5,72,82]. This pattern of expression in dry seeds is confirmed also from the RNA-seq data mining [45] presented for *M. truncatula*. 

In conclusion, this study indicates that this small plant TDP1 subfamily originated from ancient vascular plants around ~410 million years ago since both TDP1α and TDP1β sequences were found in *A. trichopoda* and *S. moellendorffii*. The gene structure and protein organization are well conserved showing the presence of the two HKD catalytic sites, along with the HIRAN motif in the case of the TDP1β sequence. The gene expression data mining comparative analysis pinpoints at tissue- and stress-specific responses. Notably, the specific expression of *TDP1β* under desiccation stress may be indicative of its involvement in desiccation tolerance, an important trait related to avoidance of cellular damage during drying and rehydration. Taken together, these results encourage further investigations into this subfamily of genes, including the unique association of HKD-HIRAN-HKD organization present in the plant TDP1β sequence, along with protein functions and implications in stress tolerance. 

## Supplementary Materials

The following are available online at https://www.mdpi.com/2073-4425/11/12/1465/s1, Table S1. Class, species and NCBI accession number of all the TDP1α sequences present in this study. Table S2. Class, species, and NCBI accession number of all the TDP1β sequences present in this study. Table S3. Accession numbers for *TDP1α* and *TDP1β* genes retrieved from Phytozome v12.1 and used to acquire the gene expression data from BAR Toronto eFP browser. Table S4. Logo sequence, E-value, number of sites and width for each motif found by MEME5.0 in TDP1α sequences. Table S5. Logo sequence, E-value, number of sites, and width for each motif found by MEME5.0 in TDP1β sequences. Figure S1. MSA extract of TDP1α HKD-I e HKD-II motifs. Figure S2. MSA extract of TDP1β HKD-I e HKD-II motifs. Figure S3. Graphical representation of densities of gaps and most conserved sites in all MSAs. Figure S4. Phylogenetic trees of TDP1α using (a) Unweighted Pair Group Method with Arithmetic mean (UPGMA) and (b) Neighbor Joining (NJ). Figure S5. Phylogenetic trees of TDP1β using (a) Unweighted Pair Group Method with Arithmetic mean (UPGMA) and (b) Neighbor Joining (NJ). Figure S6. Maximum Likelihood 500 bootstrap TDP1α tree with the respective multiple sequence alignment. Figure S7. Maximum Likelihood 500 bootstrap TDP1β tree with the respective multiple sequence alignment. Figure S8. Bayesian phylogenetic tree of TDP1 with the respective complete multiple sequence alignment.
