# Supplementary material for: Plant TDP1 (Tyrosyl-DNA Phosphodiesterase 1): A Phylogenetic Perspective and Gene Expression Data Mining
Source: Genes (Basel). 2020 Dec 7;11(12):1465. doi: 10.3390/genes11121465 (PMC7762302; doi:10.3390/genes11121465)
Supplement: Supplementary file 1 [file genes-11-01465-s001.zip › Figure_S1.pdf]

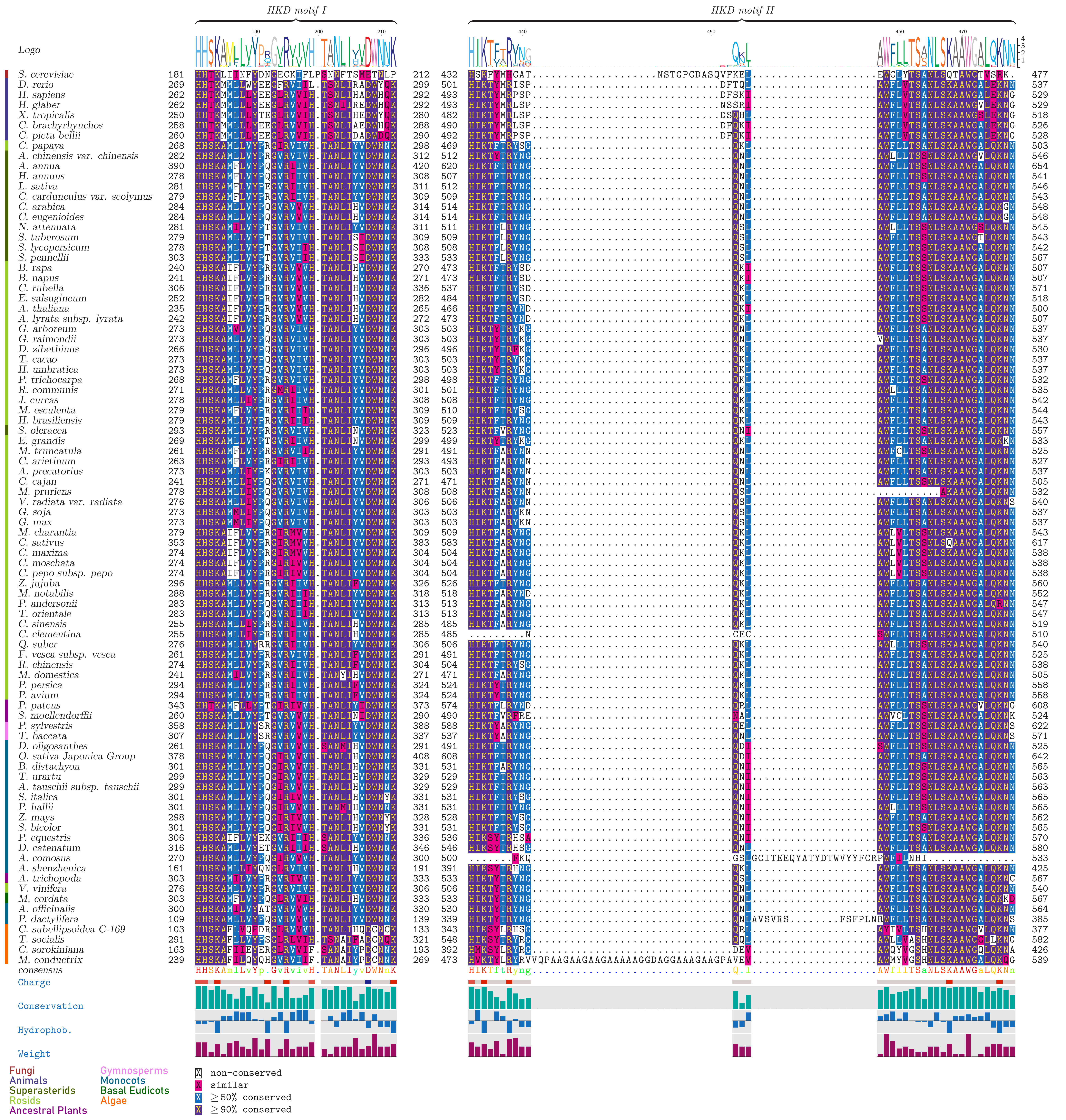

HKD motif I
HKD motif II
Logo
consensus
Charge
Conservation
Hydrophob.
Weight
Fungi
Animals
Superasterids
Rosids
Ancestral Plants
Gymnosperms
Monocots
Basal Eudicots
Algae
non-conserved
similar
≥ 50% conserved
≥ 90% conserved
