# Supplementary material for: Plant TDP1 (Tyrosyl-DNA Phosphodiesterase 1): A Phylogenetic Perspective and Gene Expression Data Mining
Source: Genes (Basel). 2020 Dec 7;11(12):1465. doi: 10.3390/genes11121465 (PMC7762302; doi:10.3390/genes11121465)
Supplement: Supplementary file 1 [file genes-11-01465-s001.zip › Figure_S2.pdf]

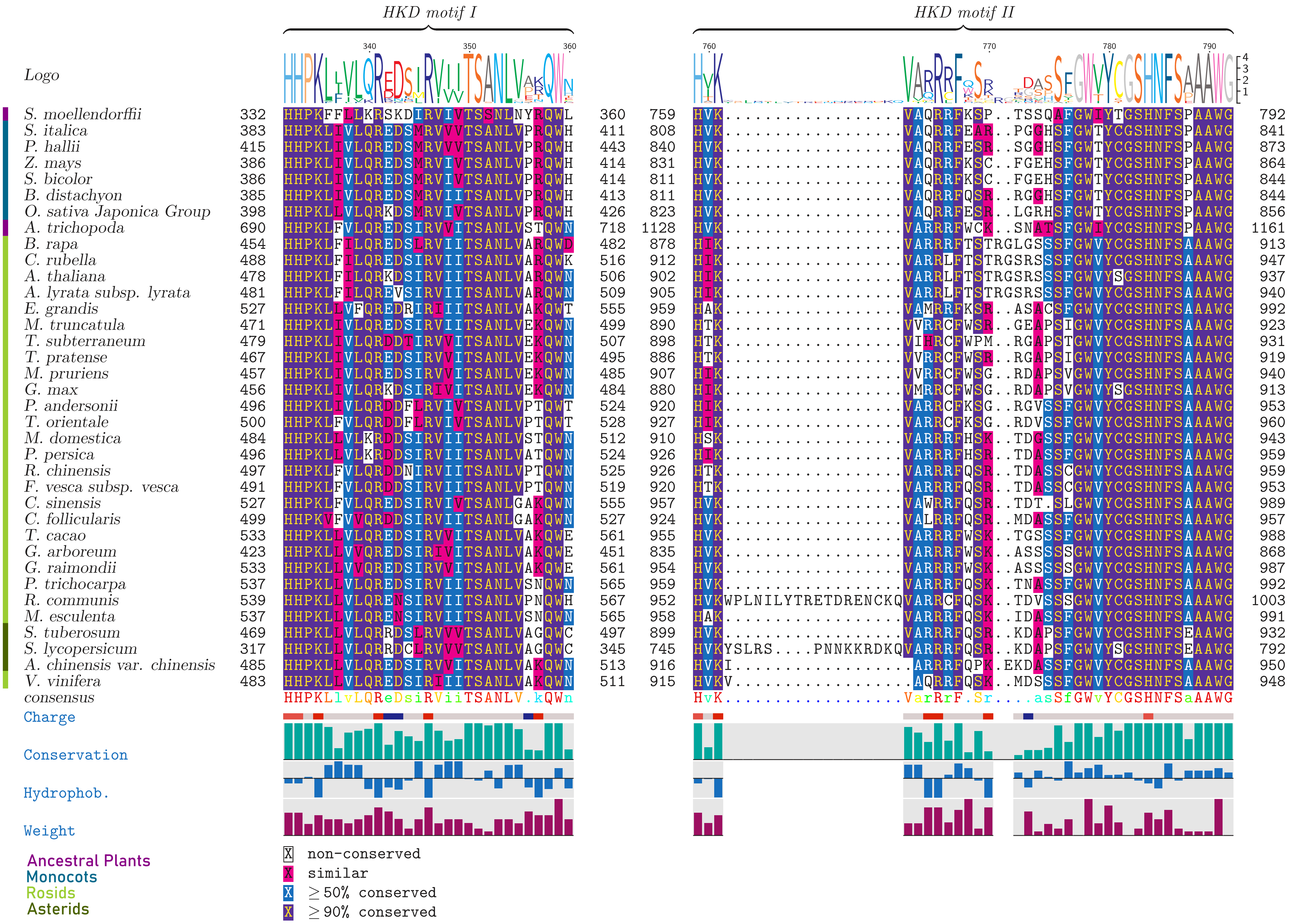

HKD motif I
HKD motif II
Logo
340
350
360
760
770
780
790
S. moellendorffii 332 360 759 792
S. italica 383 411 808 841
P. hallii 415 443 840 873
Z. mays 386 414 831 864
S. bicolor 386 414 811 844
B. distachyon 385 413 811 844
O. sativa Japonica Group 398 426 823 856
A. trichopoda 690 718 1128 1161
B. rapa 454 482 878 913
C. rubella 488 516 912 947
A. thaliana 478 506 902 937
A. lyrata subsp. lyrata 481 509 905 940
E. grandis 527 555 959 992
M. truncatula 471 499 890 923
T. subterraneum 479 507 898 931
T. pratense 467 495 886 919
M. pruriens 457 485 907 940
G. max 456 484 880 913
P. andersonii 496 524 920 953
T. orientale 500 528 927 960
M. domestica 484 512 910 943
P. persica 496 524 926 959
R. chinensis 497 525 926 959
F. vesca subsp. vesca 491 519 920 953
C. sinensis 527 555 957 989
C. follicularis 499 527 924 957
T. cacao 533 561 955 988
G. arboreum 423 451 835 868
G. raimondii 533 561 954 987
P. trichocarpa 537 565 959 992
R. communis 539 567 952 1003
M. esculenta 537 565 958 991
S. tuberosum 469 497 899 932
S. lycopersicum 317 345 745 792
A. chinensis var. chinensis 485 513 916 950
V. vinifera 483 511 915 948
consensus
HHPKLlvLQReDsiRViiTSANLV.kQWn
HvK..................VarRrF.Sr....asSfGWvYCGSHNFSaAAWG
Charge
Conservation
Hydrophob.
Weight
Ancestral Plants
Monocots
Rosids
Asterids
X non-conserved
X similar
X ≥ 50% conserved
X ≥ 90% conserved
