# Supplementary material for: Plant TDP1 (Tyrosyl-DNA Phosphodiesterase 1): A Phylogenetic Perspective and Gene Expression Data Mining
Source: Genes (Basel). 2020 Dec 7;11(12):1465. doi: 10.3390/genes11121465 (PMC7762302; doi:10.3390/genes11121465)
Supplement: Supplementary file 1 [file genes-11-01465-s001.zip › Figure_S3.pdf]

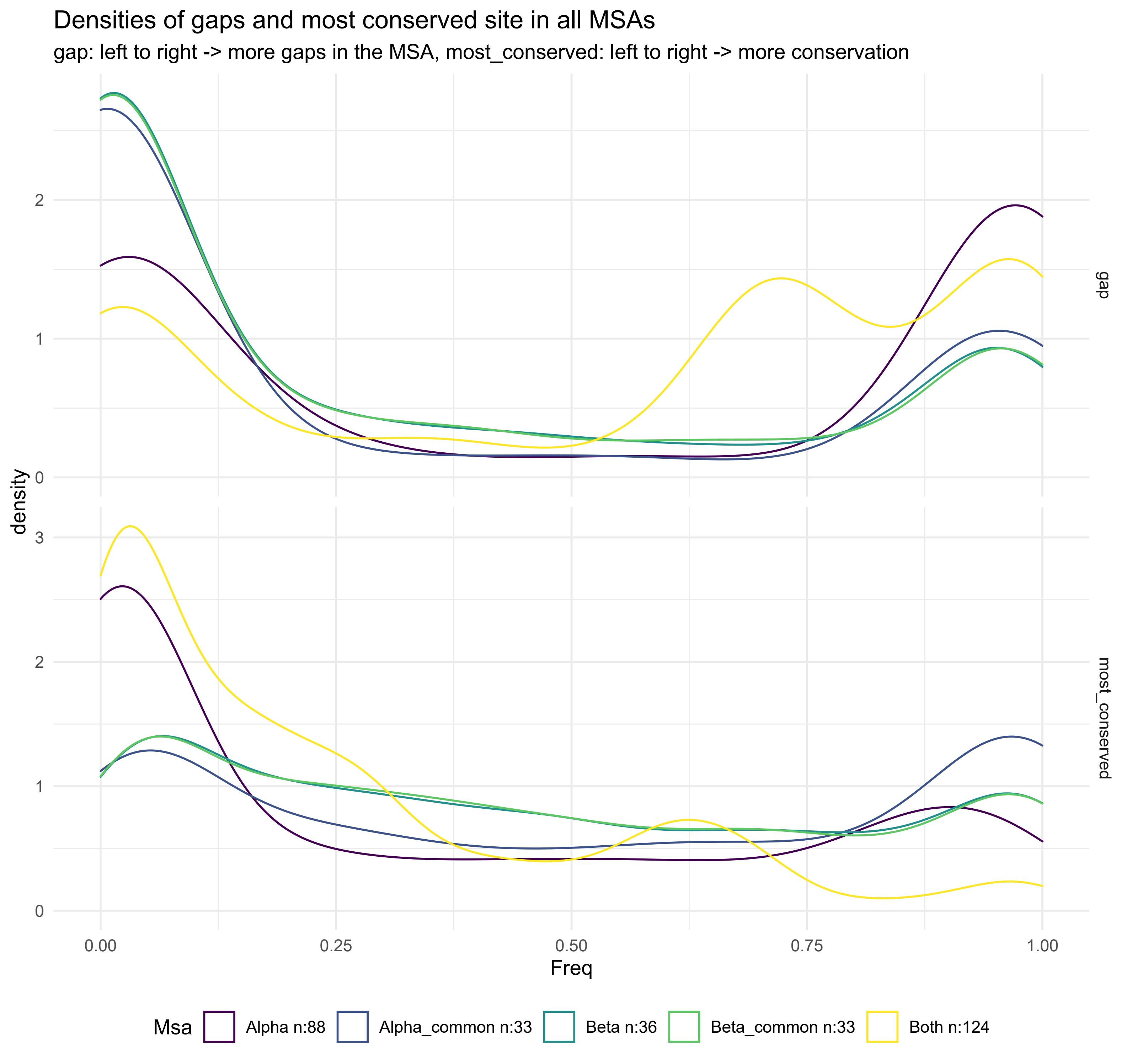
Densities of gaps and most conserved site in all MSAs
gap: left to right -> more gaps in the MSA, most_conserved: left to right -> more conservation
density
Freq
gap
most_conserved
0
1
2
3
0.00
0.25
0.50
0.75
1.00
Msa
Alpha n:88
Alpha_common n:33
Beta n:36
Beta_common n:33
Both n:124
