# Supplementary material for: Plant TDP1 (Tyrosyl-DNA Phosphodiesterase 1): A Phylogenetic Perspective and Gene Expression Data Mining
Source: Genes (Basel). 2020 Dec 7;11(12):1465. doi: 10.3390/genes11121465 (PMC7762302; doi:10.3390/genes11121465)
Supplement: Supplementary file 1 [file genes-11-01465-s001.zip › Figure_S4.pdf]

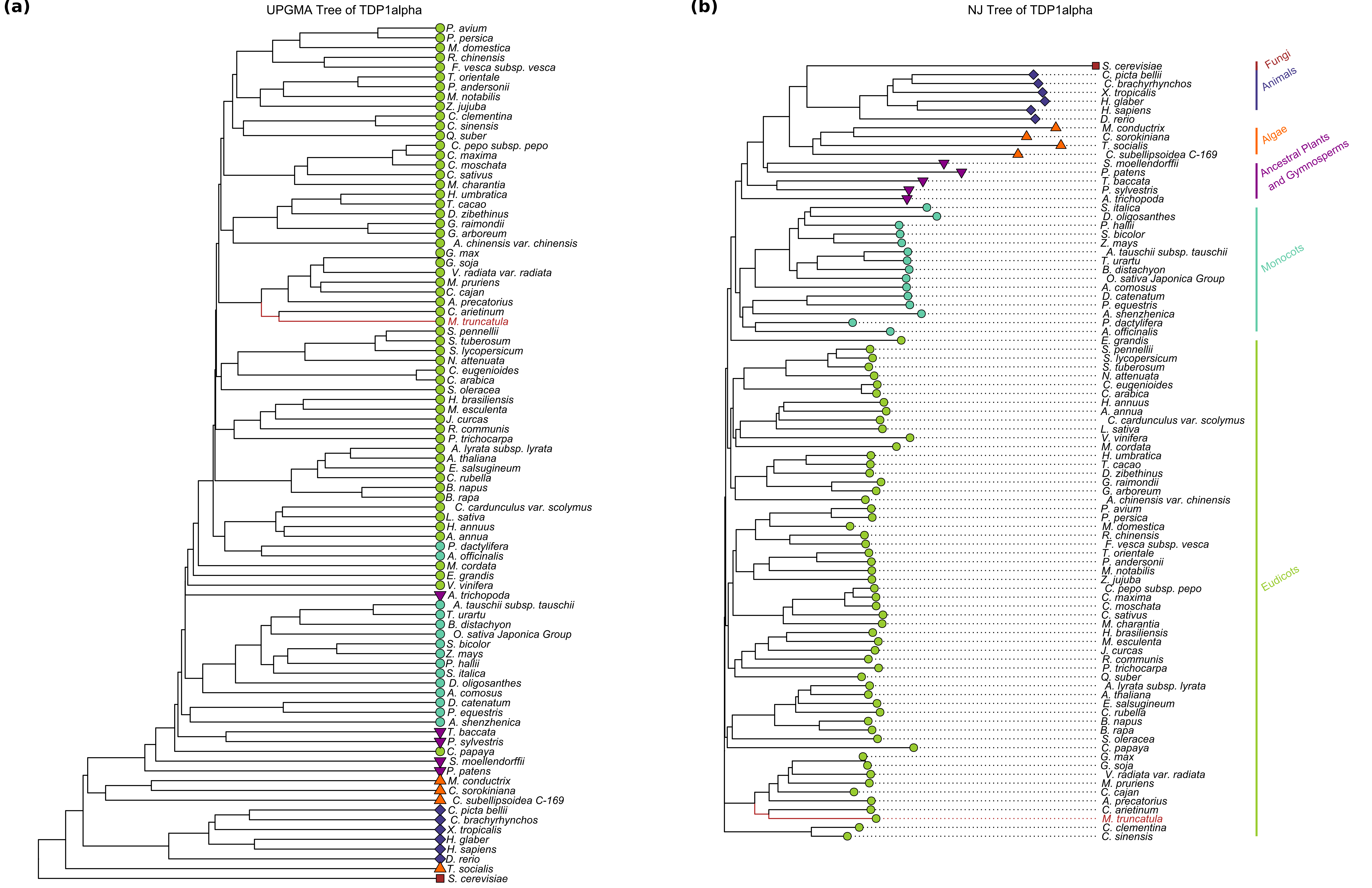
(a)
UPGMA Tree of TDP1alpha
P. avium
P. persica
M. domestica
R. chinensis
F. vesca subsp. vesca
T. orientale
P. andersonii
M. notabilis
Z. jujuba
C. clementina
C. sinensis
Q. suber
C. pepo subsp. pepo
C. maxima
C. moschata
C. sativus
M. charantia
H. umbratica
T. cacao
D. zibethinus
G. raimondii
G. arboreum
A. chinensis var. chinensis
G. max
G. soja
V. radiata var. radiata
M. pruriens
C. cajan
A. precatorius
C. arietinum
M. truncatula
S. pennellii
S. tuberosum
S. lycopersicum
N. attenuata
C. eugenioides
C. arabica
S. oleracea
H. brasiliensis
M. esculenta
J. curcas
R. communis
P. trichocarpa
A. lyrata subsp. lyrata
A. thaliana
E. salsugineum
C. rubella
B. napus
B. rapa
C. cardunculus var. scolymus
L. sativa
H. annuus
A. annua
P. dactylifera
A. officinalis
M. cordata
E. grandis
V. vinifera
A. trichopoda
A. tauschii subsp. tauschii
T. urartu
B. distachyon
O. sativa Japonica Group
S. bicolor
Z. mays
P. hallii
S. italica
D. oligosanthes
A. comosus
D. catenatum
P. equestris
A. shenzhenica
T. baccata
P. sylvestris
C. papaya
S. moellendorffii
P. patens
M. conductrix
C. sorokiniana
C. subellipsoidea C-169
C. picta bellii
C. brachyrhynchos
X. tropicalis
H. glaber
H. sapiens
D. rerio
T. socialis
S. cerevisiae
(b)
NJ Tree of TDP1alpha
S. cerevisiae
C. picta bellii
C. brachyrhynchos
X. tropicalis
H. glaber
H. sapiens
D. rerio
M. conductrix
C. sorokiniana
T. socialis
C. subellipsoidea C-169
S. moellendorffii
P. patens
T. baccata
P. sylvestris
A. trichopoda
S. italica
D. oligosanthes
P. hallii
S. bicolor
Z. mays
A. tauschii subsp. tauschii
T. urartu
B. distachyon
O. sativa Japonica Group
A. comosus
D. catenatum
P. equestris
A. shenzhenica
P. dactylifera
A. officinalis
E. grandis
S. pennellii
S. lycopersicum
S. tuberosum
N. attenuata
C. eugenioides
C. arabica
H. annuus
A. annua
C. cardunculus var. scolymus
L. sativa
V. vinifera
M. cordata
H. umbratica
T. cacao
D. zibethinus
G. raimondii
G. arboreum
A. chinensis var. chinensis
P. avium
P. persica
M. domestica
R. chinensis
F. vesca subsp. vesca
T. orientale
P. andersonii
M. notabilis
Z. jujuba
C. pepo subsp. pepo
C. maxima
C. moschata
C. sativus
M. charantia
H. brasiliensis
M. esculenta
J. curcas
R. communis
P. trichocarpa
Q. suber
A. lyrata subsp. lyrata
A. thaliana
E. salsugineum
C. rubella
B. napus
B. rapa
S. oleracea
C. papaya
G. max
G. soja
V. radiata var. radiata
M. pruriens
C. cajan
A. precatorius
C. arietinum
M. truncatula
C. clementina
C. sinensis
Fungi
Animals
Algae
Ancestral Plants
and Gymnosperms
Monocots
Eudicots
