# Supplementary material for: Plant TDP1 (Tyrosyl-DNA Phosphodiesterase 1): A Phylogenetic Perspective and Gene Expression Data Mining
Source: Genes (Basel). 2020 Dec 7;11(12):1465. doi: 10.3390/genes11121465 (PMC7762302; doi:10.3390/genes11121465)
Supplement: Supplementary file 1 [file genes-11-01465-s001.zip › Figure_S6.pdf]

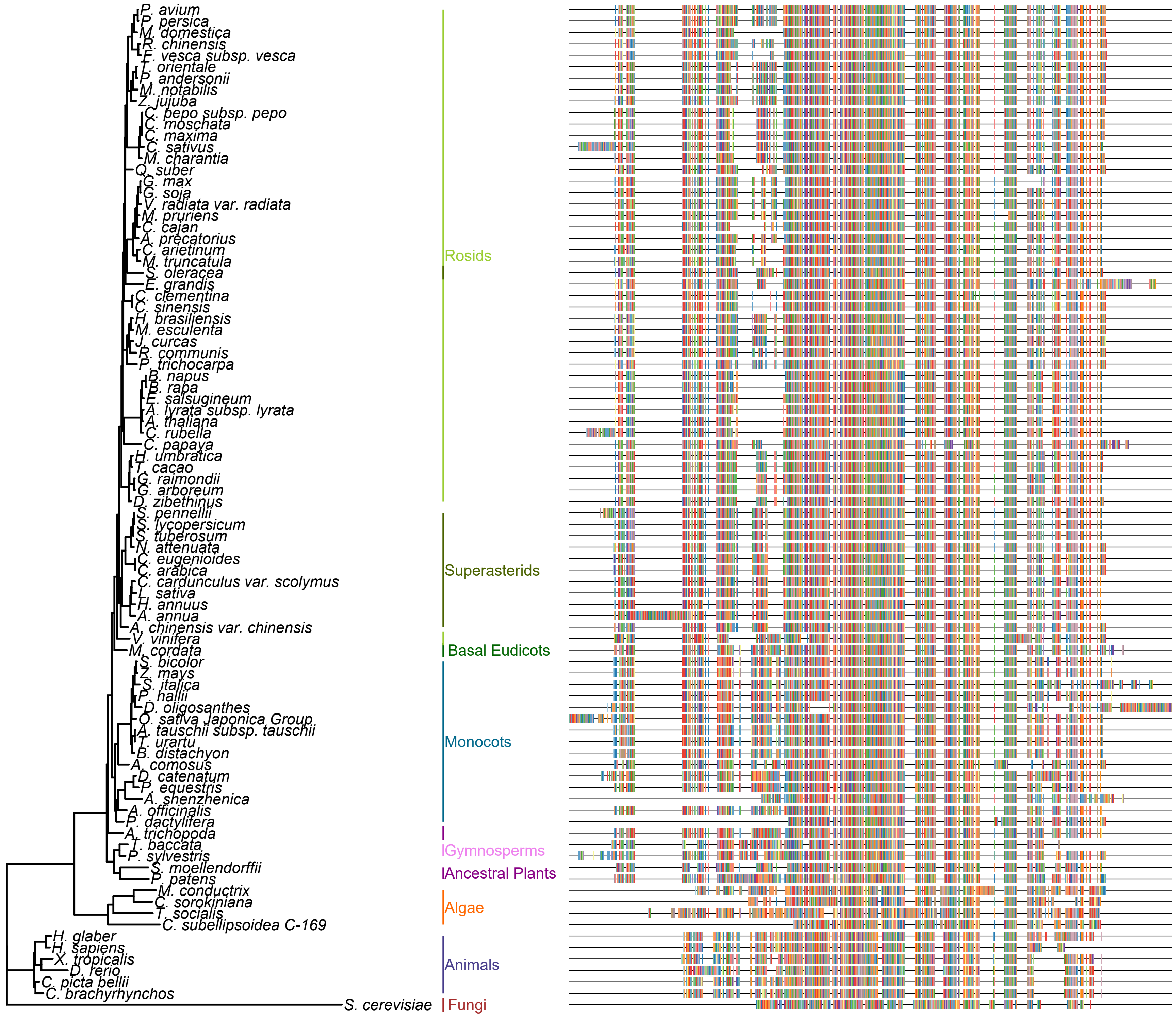
P. avium
P. persica
M. domestica
R. chinensis
F. vesca subsp. vesca
T. orientale
P. andersonii
M. notabilis
Z. jujuba
C. pepo subsp. pepo
C. moschata
C. maxima
C. sativus
M. charantia
Q. suber
G. max
G. soja
V. radiata var. radiata
M. pruriens
C. cajan
A. precatorius
C. arietinum
M. truncatula
S. oleracea
E. grandis
C. clementina
C. sinensis
H. brasiliensis
M. esculenta
J. curcas
R. communis
P. trichocarpa
B. napus
B. rapa
E. salsugineum
A. lyrata subsp. lyrata
A. thaliana
C. rubella
C. papaya
H. umbratica
T. cacao
G. raimondii
G. arboreum
D. zibethinus
S. pennellii
S. lycopersicum
S. tuberosum
N. attenuata
C. eugenioides
C. arabica
C. cardunculus var. scolymus
L. sativa
H. annuus
A. annua
A. chinensis var. chinensis
V. vinifera
M. cordata
S. bicolor
Z. mays
S. italica
P. hallii
D. oligosanthes
O. sativa Japonica Group
A. tauschii subsp. tauschii
T. urartu
B. distachyon
A. comosus
D. catenatum
P. equestris
A. shenzhenica
A. officinalis
P. dactylifera
A. trichopoda
T. baccata
P. sylvestris
S. moellendorffii
P. patens
M. conductrix
C. sorokiniana
T. socialis
C. subellipsoidea C-169
H. glaber
H. sapiens
X. tropicalis
D. rerio
C. picta bellii
C. brachyrhynchos
S. cerevisiae
Rosids
Superasterids
Basal Eudicots
Monocots
Gymnosperms
Ancestral Plants
Algae
Animals
Fungi
