# Supplementary material for: Plant TDP1 (Tyrosyl-DNA Phosphodiesterase 1): A Phylogenetic Perspective and Gene Expression Data Mining
Source: Genes (Basel). 2020 Dec 7;11(12):1465. doi: 10.3390/genes11121465 (PMC7762302; doi:10.3390/genes11121465)
Supplement: Supplementary file 1 [file genes-11-01465-s001.zip › Tables.pdf]

Table S1. Class, species and NCBI accession number of all the TDP1α sequences present in this study.

Class	Species	NCBI Accession
Eudicots	*M. truncatula*	XP_003622687
Eudicots	*C. arietinum*	XP_012568966
Eudicots	*A. precatorius*	XP_027361208
Eudicots	*C. cajan*	KYP67403
Eudicots	*G. max*	XP_006578130
Eudicots	*G. soja*	KHN29597
Eudicots	*V. radiata*	XP_014520570
Eudicots	*M. pruriens*	RDX62056
Eudicots	*Q. suber*	XP_023920145
Eudicots	*C. clementina*	XP_024046807
Eudicots	*C. sinensis*	XP_006467985
Eudicots	*M. esculenta*	XP_021599995
Eudicots	*T. orientale*	PON85342
Eudicots	*J. curcas*	XP_012091409
Eudicots	*H. brasiliensis*	XP_021642094
Eudicots	*M. notabilis*	EXB31386
Eudicots	*V. vinifera*	RVW45926
Eudicots	*P. andersonii*	PON39438
Eudicots	*P. avium*	XP_021821508
Eudicots	*S. oleracea*	XP_021836412
Eudicots	*G. arboreum*	KHG26217
Eudicots	*P. persica*	XP_020416746
Eudicots	*R. communis*	XP_015574204
Eudicots	*C. eugenioides*	XP_027163996
Eudicots	*R. chinensis*	XP_024159806
Eudicots	*C. arabica*	XP_027105045
Eudicots	*Z. jujuba*	XP_015883785
Eudicots	*A. chinensis*	PSS04715
Eudicots	*H. umbratica*	XP_021294271
Eudicots	*C. pepo*	XP_023544483
Eudicots	*C. moschata*	XP_022950185
Eudicots	*S. pennellii*	XP_015067766
Eudicots	*C. maxima*	XP_022978030
Eudicots	*N. attenuata*	OIT34941
Eudicots	*C. cardunculus*	XP_024983894
Eudicots	*S. lycopersicum*	XP_010317667
Eudicots	*D. zibethinus*	XP_022720581
Eudicots	*P. trichocarpa*	XP_006383945
Eudicots	*M. charantia*	XP_022142241
Eudicots	*H. annuus*	XP_022015799
Eudicots	*L. sativa*	XP_023743532
Eudicots	*A. lyrata*	XP_020879079
Eudicots	*B. napus*	XP_013728150
Eudicots	*E. salsugineum*	XP_006400034

Table S1. *Cont.*

Class	Species	NCBI Accession
Eudicots	*A. annua*	PWA63244
Eudicots	*M. cordata*	OVA11144
Eudicots	*A. thaliana*	ACO60340
Eudicots	*C. rubella*	XP_006290125
Eudicots	*M. domestica*	XP_008384030
Eudicots	*F. vesca*	XP_011459955
Eudicots	*B. rapa*	XP_009131446
Eudicots	*C. papaya*	XP_021887357
Eudicots	*G. raimondii*	XP_012456873
Eudicots	*T. cacao*	XP_007025983
Eudicots	*E. grandis*	XP_010052433
Eudicots	*S. tuberosum*	XP_006343414
Eudicots	*C. sativus*	XP_011658164
Lycophita	*S. moellendorffii*	XP_002970738
Bryophyta	*P. patens*	XP_024385825
Ancestral	*A. trichopoda*	XP_020517687
Monocots	*A. comosus*	OAY67291
Monocots	*P. hallii*	XP_025800030
Monocots	*S. italica*	XP_022683988
Monocots	*A. officinalis*	XP_020256587
Monocots	*T. urartu*	EMS46186
Monocots	*S. bicolor*	XP_021309892
Monocots	*D. catenatum*	XP_020688319
Monocots	*Z. mays*	XP_008651694
Monocots	*A. tauschii*	XP_020162379
Monocots	*B. distachyon*	XP_024313659
Monocots	*P. equestris*	XP_020589644
Monocots	*O. sativa*	XP_015646620
Monocots	*P. dactylifera*	XP_026664181
Monocots	*A. shenzhenica*	PKA57825
Monocots	*D. oligosanthes*	OEL28790
Gymnosperm	*P. sylvestris*	PSY00009297
Gymnosperm	*T. baccata*	TBA00005746
Algae	*C. sorokiniana*	PRW59620
Algae	*M. conductrix*	PSC74986
Algae	*C. subellipsoidea*	EIE25781
Algae	*T. socialis*	PNH10367
Fungi	*S. cerevisiae*	KZV13308
Animals	*H. glaber*	EHB09657
Animals	*H. sapiens*	AAI07878
Animals	*C. picta bellii*	XP_008163939
Animals	*D. rerio*	XP_700174
Animals	*X. tropicalis*	CAJ81457
Animals	*C. brachyrhynchos*	KFO56089

Table S2. Class, species and NCBI accession number of all the TDP1β sequences present in this study.

Class	Species	NCBI Accession
Eudicots	*M. truncatula*	AET04910
Eudicots	*T. pratense*	PNY07045
Eudicots	*M. pruriens*	RDX75863
Eudicots	*T. orientale*	PON97503
Eudicots	*P. andersonii*	PON71257
Eudicots	*A. chinensis*	PSR89388
Eudicots	*G. arboreum*	KHG24729
Eudicots	*R. chinensis*	PRQ16808
Eudicots	*C. follicularis*	GAV73409
Eudicots	*R. communis*	EEF51660
Eudicots	*T. subterraneum*	GAU25446
Eudicots	*M. esculenta*	XP_021614780
Eudicots	*P. trichocarpa*	XP_024438691
Eudicots	*G. max*	XP_006584680
Eudicots	*P. persica*	XP_020418919
Eudicots	*M. domestica*	XP_008356812
Eudicots	*F. vesca*	XP_011470841
Eudicots	*A. thaliana*	OAO91221
Eudicots	*A. lyrata*	XP_020876114
Eudicots	*C. rubella*	XP_023637563
Eudicots	*B. rapa*	XP_009125687
Eudicots	*G. raimondii*	XP_012467165
Eudicots	*T. cacao*	XP_007038197
Eudicots	*C. sinensis*	XP_006485048
Eudicots	*E. grandis*	XP_010065187
Eudicots	*V. vinifera*	XP_010644043
Eudicots	*S. tuberosum*	XP_006341694
Eudicots	*S. lycopersicum*	XP_025885954
Monocots	*S. bicolor*	XP_002446388
Monocots	*Z. mays*	ONM62577
Monocots	*S. italica*	RCV33451
Monocots	*P. halli*	XP_025826012
Monocots	*O. sativa*	XP_015635430
Monocots	*B. distachyon*	XP_010239843
Lycophita	*S. moellendorffii*	XP_002993511
Ancestral	*A. trichopoda*	XP_011623559

Table S3. Accession numbers for *TDP1α* and *TDP1β* genes retrieved from Phytozome v12.1 (https://phytozome.jgi.doe.gov/) and used to acquire the gene expression data from BAR Toronto eFP browser (http://bar.utoronto.ca/eplant).

**Species**	***TDP1α***	***TDP1β***
Selaginella moellendorffii	Smo170833	Smo415650
Zea mays	GRMZM2G080174	GRMZM2G069856
Oryza sativa	LOC_Os07g34598	LOC_Os04g33050
Medicago truncatula	Medtr7g050860	Medtr8g095490
Glycine max	Glyma.04G064300	Glyma.08G005800
Solanum lycopersicum	Solyc03g031820.2	Solyc03g117960.2
Solanum tuberosum	PGSC0003DMG400024073	PGSC0003DMG400014226

Table S4. Logo sequence, E-value, number of sites and width for each motif found by MEME5.0 in TDP1α sequences.

Motifs TDP1 alpha	E-value	Sites	Width
1. 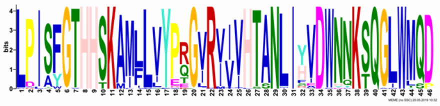	3.8e-3023	69	46
2. 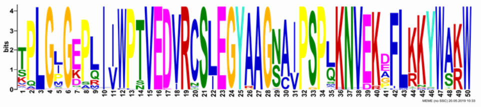	4.7e-2742	61	50
3. 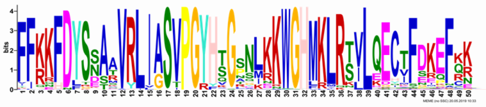	6.3e-2726	69	50
4. 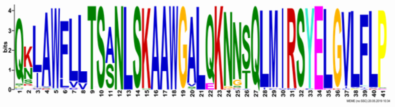	6.8e-2648	69	41
5. 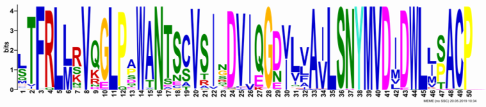	1.1e-2634	62	50
6. 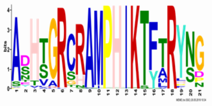	8.0e-1196	68	21

Table S5. Logo sequence, E-value, number of sites and width for each motif found by MEME5.0 in TDP1β sequences.

Motifs TDP1 beta	E-value	Sites	Width
1. 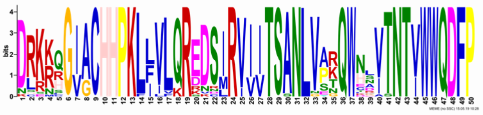	1.1e-1062	26	50
2. 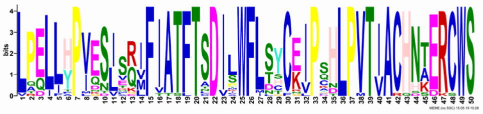	1.4e-981	26	50
3. 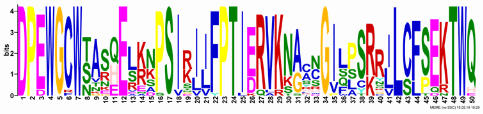	6.1e-917	26	50
4. 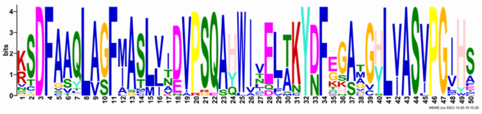	1.0e-874	26	50
5. 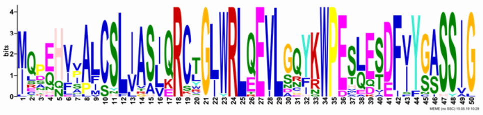	9.4e-849	26	50
6. 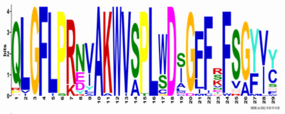	2.0e-516	26	29
7. 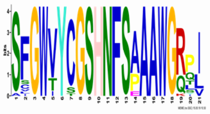	1.7e-436	26	21
