# Supplementary figures and images for: Plant TDP1 (Tyrosyl-DNA Phosphodiesterase 1): A Phylogenetic Perspective and Gene Expression Data Mining
Source: Genes (Basel). 2020 Dec 7;11(12):1465. doi: 10.3390/genes11121465 (PMC7762302; doi:10.3390/genes11121465)

**(a)**

UPGMA Tree of TDP1beta

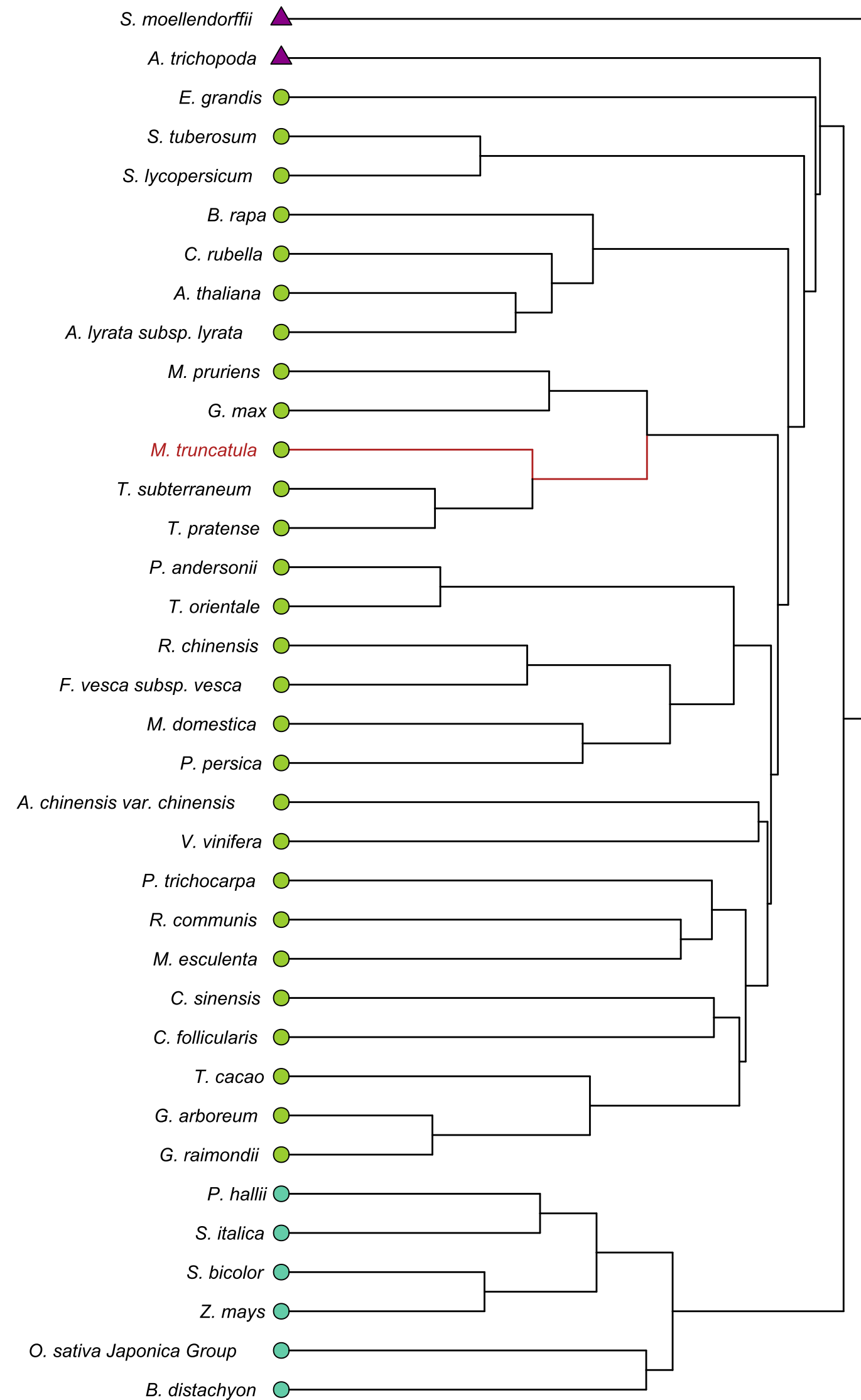**(b)**

NJ Tree of TDP1beta

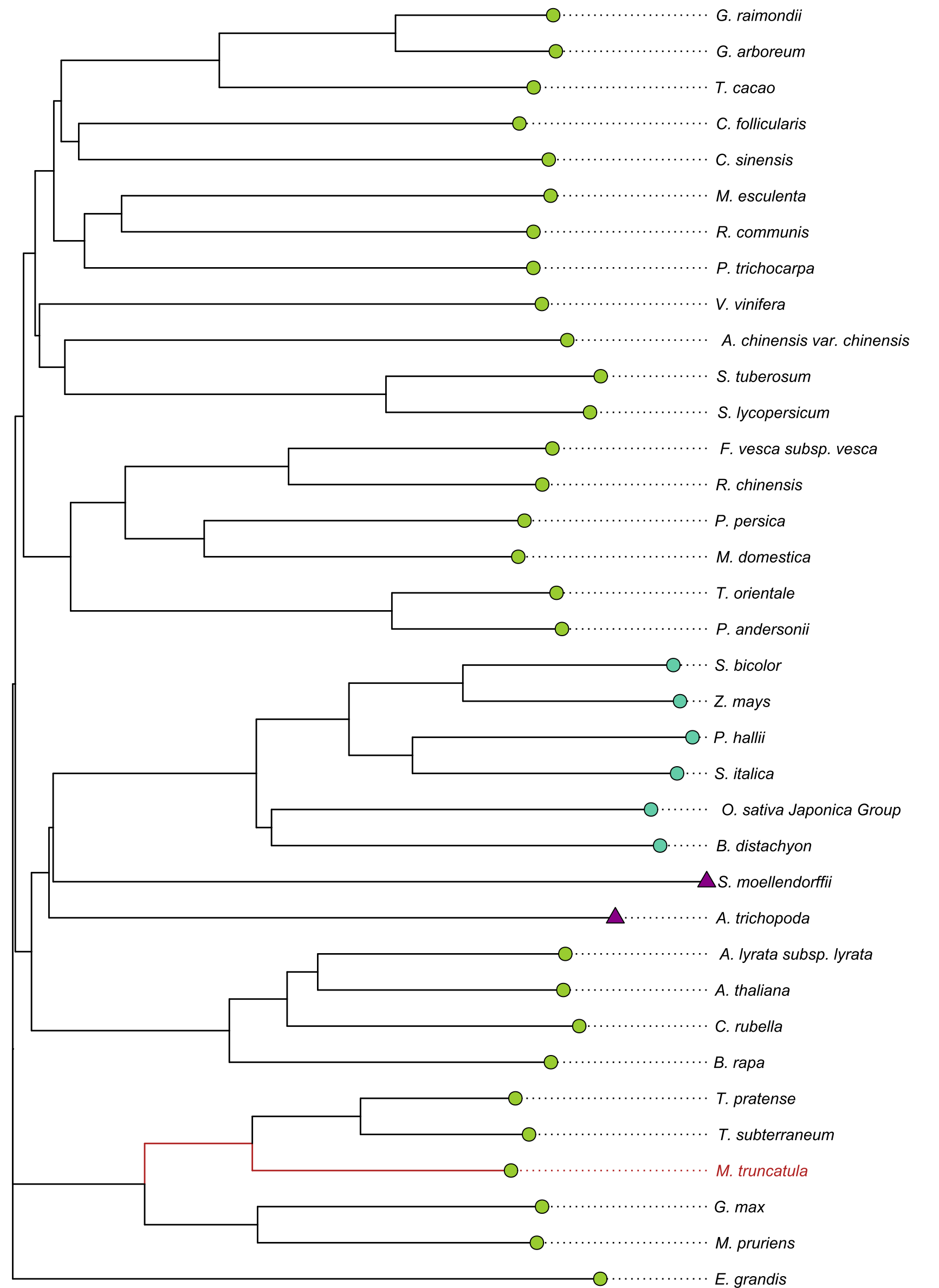

Supplement: Supplementary file 1 [file genes-11-01465-s001.zip › Figure_S5.pdf]

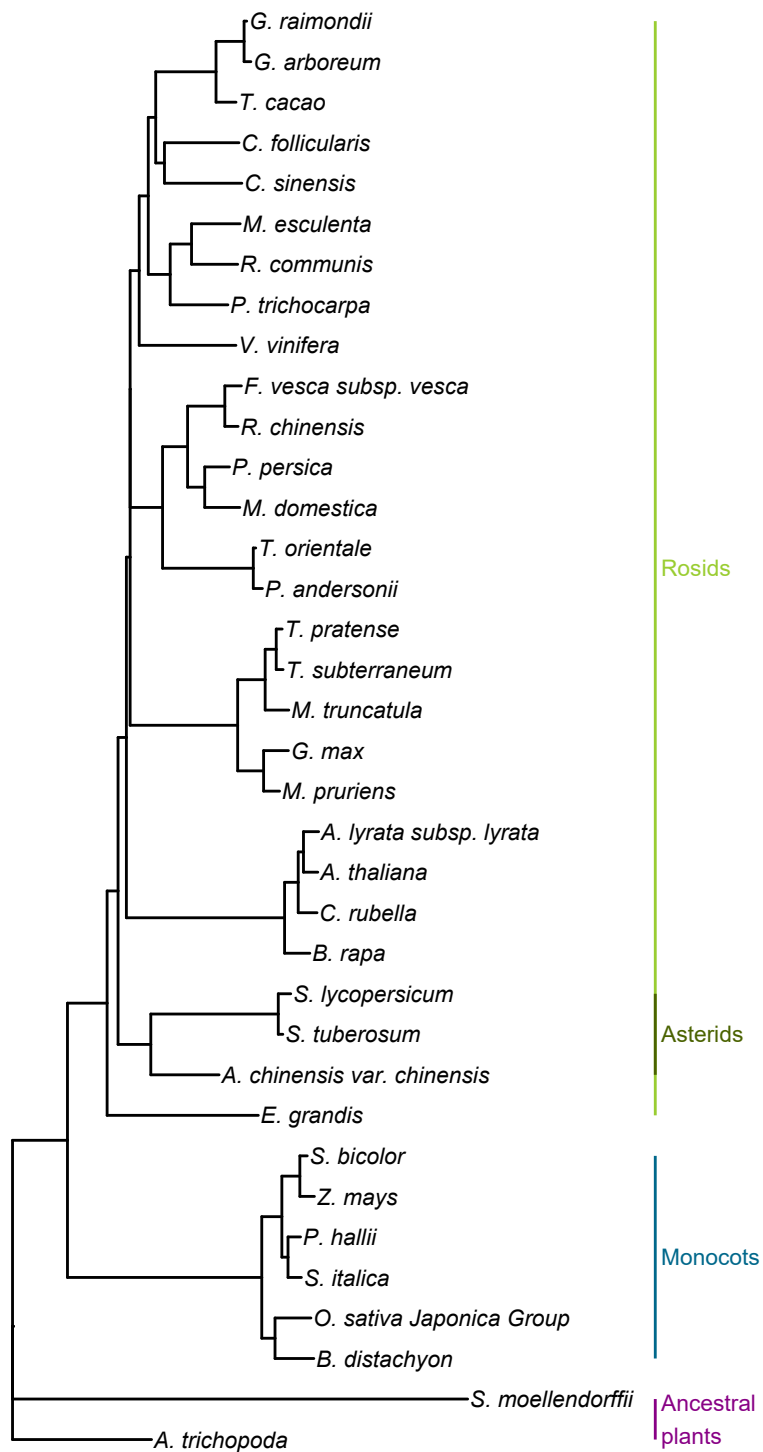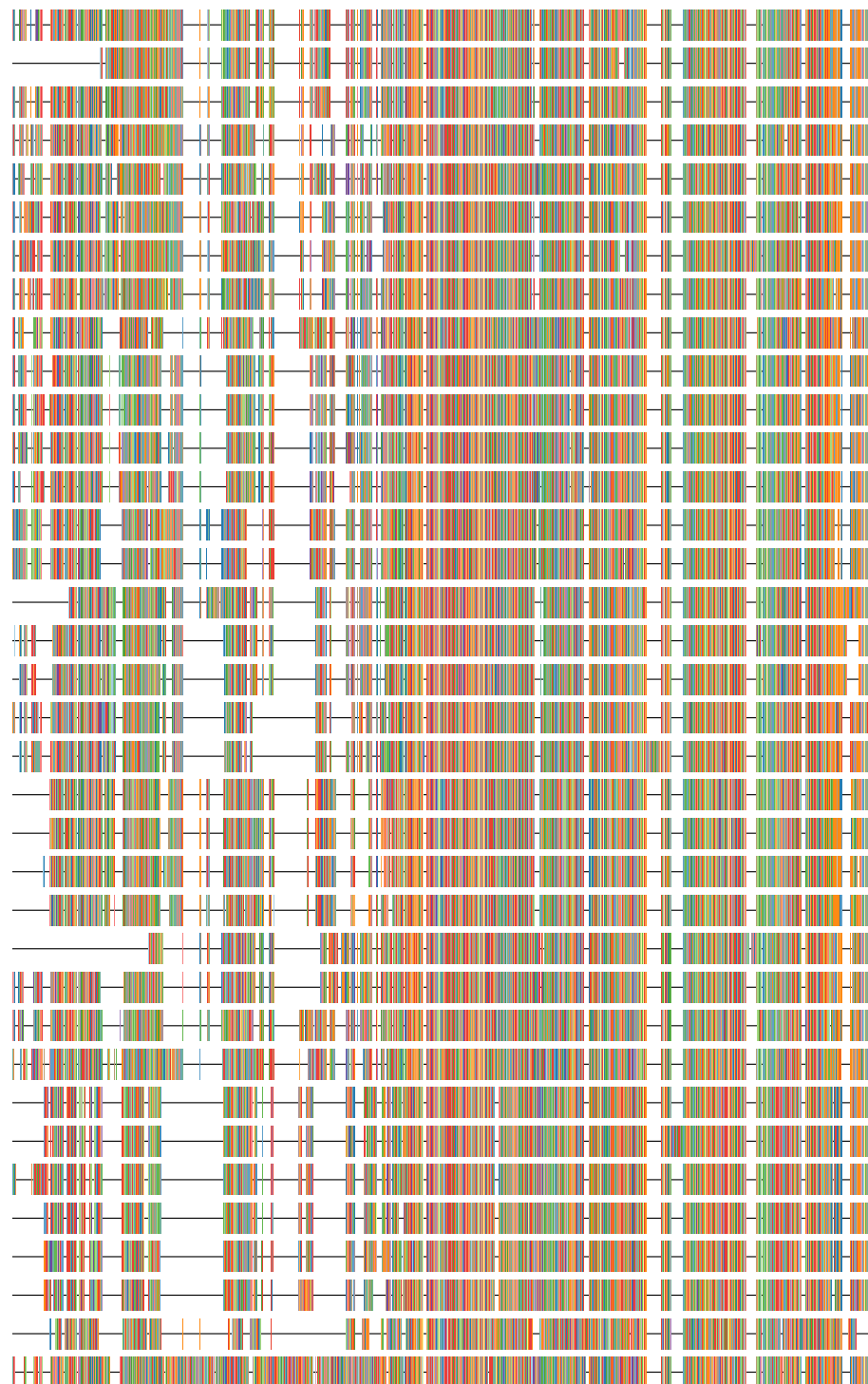

Supplement: Supplementary file 1 [file genes-11-01465-s001.zip › Figure_S7.pdf]

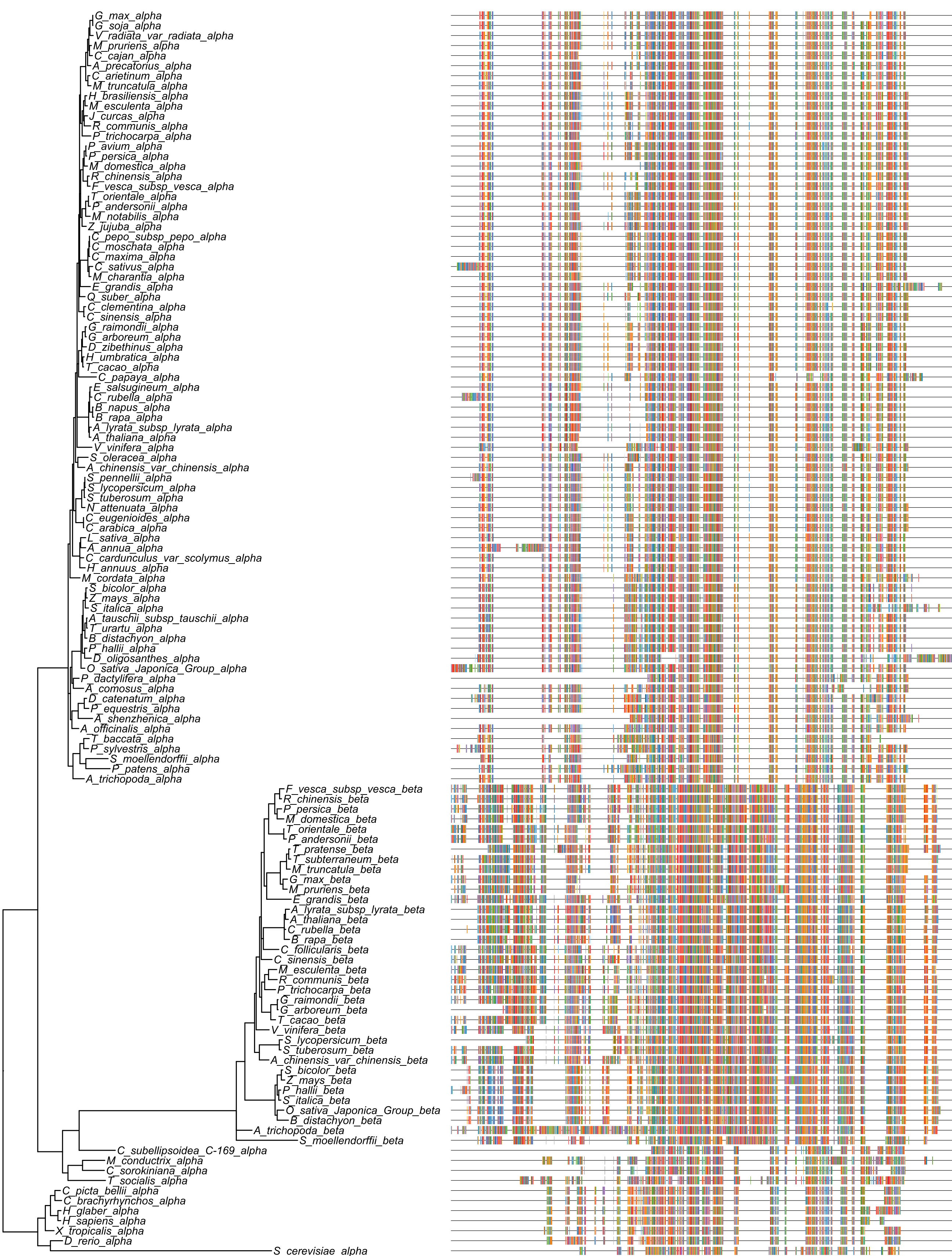

Supplement: Supplementary file 1 [file genes-11-01465-s001.zip › Figure_S8.pdf]
